# Blood-based epigenetic biomarkers in rheumatoid arthritis: current knowledge and future perspectives

**DOI:** 10.3389/fimmu.2026.1753808

**Published:** 2026-02-20

**Authors:** Agnieszka Mołoń, Hubert Kubis, Joanna Żurawska, Marek Cieśla

**Affiliations:** Laboratory of Diagnostic and Clinical Epigenetics Faculty of Medicine, Collegium Medicum, University of Rzeszów, Rzeszów, Poland

**Keywords:** DNA methylation, epigenetics, histone modifications, non-coding RNAs, peripheral blood biomarkers, rheumatoid arthritis, RNA methylation

## Abstract

Rheumatoid arthritis (RA) is a chronic systemic autoimmune disease that leads to progressive joint destruction, extra-articular manifestations, disability, and increased mortality. Early detection, particularly in seronegative patients, remains challenging because current diagnostic criteria based on joint involvement, serology, and acute-phase reactants may fail to identify disease at its earliest stages. Epigenetic mechanisms, including DNA and RNA methylation, histone modifications, and non-coding RNAs (ncRNAs), provide a dynamic interface between genetic predisposition and environmental triggers in RA pathogenesis. Peripheral blood (plasma, serum, and cellular fractions) is an accessible, minimally invasive source for monitoring systemic molecular alterations over time. To capture the latest evidence, we performed a structured literature search using curated keywords covering RA, epigenetic mechanisms, DNA and RNA methylation, m6A, histone modifications, miRNAs, lncRNAs, circRNAs, and blood-based fractions (peripheral blood mononuclear cells (PBMCs), plasma, serum, whole blood). Emerging data indicate that blood-based epigenetic signatures not only reflect disease activity but also hold promise as prognostic biomarkers, predictors of treatment response, and tools for personalized therapeutic strategies. In this review, we synthesize current knowledge on blood-based epigenetic alterations in RA, focusing on DNA methylation, histone modifications, and multiple classes of ncRNAs, including less widely studied species such as piRNAs, snoRNAs, Y-RNAs, snRNAs, and tRNA-derived fragments, with an emphasis on studies published between 2020 and 2025. We highlight the translational potential of multilayered epigenetic signatures as innovative diagnostic and prognostic tools that could advance early detection and guide precision-medicine approaches in RA.

## Graphical Abstract

**Figure:**
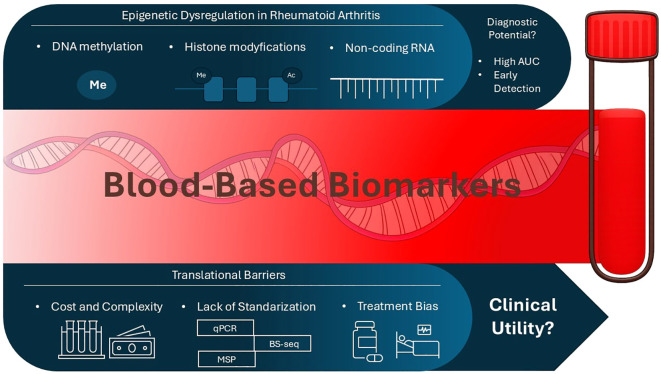

## 1 Introduction

Rheumatoid arthritis (RA) is a chronic, systemic autoimmune disease that leads to progressive disability and systemic manifestations, ultimately resulting in premature mortality (1, 2). The inflammatory process typically begins in smaller joints, presenting with discomfort, stiffness, and pain. As the disease progresses, it involves larger joints, impairs their function, and may lead to severe disability (3). In extreme cases, extra-articular manifestations may occur, affecting the skin, eyes, lungs, and heart, potentially contributing to premature death (1). RA affects approximately 1% of the global population (4), with a higher prevalence in women than men, and the peak incidence occurring between 40 and 50 years of age (5). The disease develops as a result of complex interactions among genetic, environmental, and immunological factors. Among the most significant genetic predispositions is carriage of the HLA-DRB1 allele, referred to as the “shared epitope” which markedly increases the risk of developing RA, although its presence alone is not sufficient to trigger disease onset. Environmental factors, such as tobacco smoking, infections, and oxidative stress, act as additional stimuli initiating the pathological inflammatory process (6). The pathogenesis of RA is driven by aberrant activation of T and B lymphocytes, resulting in the production of autoantibodies, including rheumatoid factor (RF) and anti-citrullinated protein antibodies (ACPA, anti-CCP). This is accompanied by increased production of pro-inflammatory cytokines, such as TNF-α, IL-6, and IL-1β, which mediate inflammation, joint tissue destruction, pain, and functional impairment (2). Despite advances in understanding the immunological mechanisms underlying RA, the phenotypic variability and heterogeneity of its clinical course suggest the presence of additional disease-modifying factors.

Currently, the diagnosis of RA is based on the 2010 classification criteria established by the American College of Rheumatology and the European League Against Rheumatism (ACR/EULAR). These criteria encompass four principal diagnostic domains: the number of affected joints, serology (including levels of RA-associated autoantibodies such as RF and ACPA), acute-phase reactants (APRs), including elevated C-reactive protein (CRP) and erythrocyte sedimentation rate (ESR), and the duration of symptoms (7). The implementation of the 2010 criteria has significantly improved disease detection, particularly in early-stage arthritis, compared to previously used criteria (8–10).

The presence of autoantibodies, including ACPA and RF, provides a robust foundation for diagnostic classification. However, a substantial subset of RA patients remains ACPA-negative, indicating the existence of disease subtypes with distinct immunological profiles and potentially differing pathogenic mechanisms (11). Moreover, approximately 10% of all RA patients are not identified at early stages of the disease, of whom nearly 9% are seronegative, despite the critical importance of early diagnosis for both seropositive and seronegative patients (12–15). The integration of innovative diagnostic biomarkers may enhance detection efficacy, particularly in scenarios where conventional classification criteria are insufficient, such as in seronegative patients. Despite the growing body of evidence, there remains a lack of consistent and clinically validated epigenetic biomarkers derived from peripheral blood that could be implemented in routine clinical practice. Increasing attention is being directed toward epigenetic mechanisms, which act as a bridge between genotype and environmental influences. Epigenetics encompasses gene expression modifications independent of DNA sequence changes, including DNA methylation, histone modifications, and regulation by non-coding RNAs (ncRNAs) in RA (2, 16, 17).

Peripheral blood provides a practical and clinically relevant biological material for studying systemic molecular alterations associated with RA. It is easily accessible, minimally invasive, and suitable for repeated sampling, enabling longitudinal monitoring of disease-related molecular dynamics. Fractionation of blood into plasma, serum, and cellular components facilitates standardized analysis and supports the detection of circulating biomarkers, including stable extracellular ncRNAs protected within vesicles or ribonucleoprotein complexes (18–20).

Analyses of peripheral blood allow for the assessment of systemic molecular changes that may reflect early disease stages before the onset of clinical joint symptoms. The presence of ACPA in serum long before clinical manifestations suggests that RA pathogenesis may begin systemically prior to localization in the joints. This challenges the traditional view of RA as a disease initially confined to synovial tissue. Serum ACPA thus exemplifies a biomarker that can signal systemic alterations preceding local joint damage (21).

The aim of this review is to provide an integrated overview of epigenetic alterations in rheumatoid arthritis, encompassing DNA methylation, histone modifications, and non-coding RNA-mediated regulation, with a particular focus on blood-based biomarkers. We critically evaluate their clinical performance, reproducibility, and translational feasibility for diagnostic and prognostic applications. Special emphasis is placed on peripheral blood and its fractions as accessible biospecimens, as well as on emerging classes of non-coding RNAs that may expand current biomarker and therapeutic paradigms in RA.

## 2 Methods

### 2.1 Literature search strategy

This narrative review was conducted using a structured and reproducible literature search strategy designed to capture both molecular and clinical aspects of rheumatoid arthritis (RA), with a specific focus on blood-based epigenetic biomarkers. Bibliographic searches were performed in PubMed/MEDLINE, Web of Science Core Collection, and Scopus. To ensure comprehensive coverage, database searches were complemented by manual screening of reference lists from relevant original articles and reviews. A predefined set of keywords was used alone and in combination, including: *“rheumatoid arthritis”, “RA”, “epigenetic”, “DNA methylation”, “RNA methylation”, “m6A”, “histone modifications”, “miRNA”, “lncRNA”, “circRNA”, “ncRNA”, “peripheral blood mononuclear cells (PBMCs)”, “serum”, “plasma”, and “whole blood”*. Search terms were selected to encompass the full spectrum of epigenetic mechanisms and circulating biomarkers relevant to RA. The search was restricted to English-language articles published between January 2020 and September 2025, to ensure methodological consistency and focus on contemporary epigenetic technologies.

### 2.2 Eligibility criteria

Studies were eligible for inclusion if they met all of the following criteria:

1. Conducted in human populations diagnosed with RA according to established clinical criteria (e.g., ACR/EULAR).
2. Analyzed blood-based material, including whole blood, plasma, serum, PBMCs, or defined immune cell subsets.
3. Employed validated epigenetic methodologies, such as quantitative methylation-specific PCR, bisulfite sequencing, RNA sequencing, microarrays, chromatin immunoprecipitation sequencing (ChIP-seq), or ATAC-seq.
4. Included a control group (healthy individuals and/or patients with other rheumatic diseases).
5. Reported diagnostic or prognostic performance metrics (e.g., sensitivity, specificity, ROC AUC) and/or provided mechanistic insights relevant to RA pathogenesis.

Studies were excluded if they were based exclusively on animal models or *in vitro* systems, lacked a control group, were case reports, conference abstracts, editorials, or narrative reviews without original data or did not provide sufficient methodological detail regarding epigenetic assessment.

### 2.3 Study selection and data extraction

Study selection was performed in multiple stages. Initially, three independent reviewers screened titles and abstracts to identify potentially relevant publications. Full texts of eligible or uncertain records were subsequently assessed against the predefined inclusion and exclusion criteria. Discrepancies were resolved through discussion and consensus. For included studies, data were extracted regarding study design and population, biological material analyzed, epigenetic targets and analytical methodologies, reported diagnostic or prognostic metrics, and key limitations affecting translational interpretation. The present work represents a structured narrative synthesis of the literature and does not include a formal quantitative meta-analysis.

### 2.4 Clinical trial registry search

To identify ongoing or completed clinical studies addressing epigenetic mechanisms in RA, an additional search was performed in ClinicalTrials.gov using the keyword *“rheumatoid arthritis”* (last accessed September 2025). Trials were screened to identify studies explicitly investigating epigenetic modifications, epigenetic regulators, or epigenetic biomarkers, including DNA or RNA methylation, histone modifications, miRNAs, or other non-coding RNAs. Trials not directly related to RA, withdrawn prior to enrolment, or lacking epigenetic endpoints were excluded. This process identified seven relevant clinical trials, primarily focused on miRNA modulation in response to therapy, as well as one study investigating a selective HDAC6 inhibitor (summarized in **Table 1**).

**Table 1:** A literature review on DNA methylation changes involved in RA, investigated in blood and its fractions.

Studied changes	Cell/tissue type	Direction of methylation change	Biological function/effect	Clinical comparison	ROC, AUC	Sample size/key limitations	Reference
UBASH3A	CD4^+^ T cells	↓	Negative regulator of NF-kB signaling in T cells on stimulation of the antigen T cell receptor	RA MTX treated in remission vs HC	n/a	N = 18 RA/9 HC; prospective study, medications/unequal statistical power may influence results; reduced representation bisulfite sequencing	(227)
RUFY1	CD4^+^ lymphocytes	↓	Modulation of macrophage inflammatory response, induction of pro-inflammatory cytokine production, alteration of endosome function in synovial cells	RA prior to therapeutic immune modulation vs HC	n/a	N= 45 RA/64 HC; prospective study, cell subset-specific DNA method	(228)
PADI4	Whole blood	↓	Participates in protein citrullination and ACPA formation	RA vs HC	n/a	N= 125 RA, 30 HC; prospective study, heterogeneous; RA patients in various stages of the disease; qPCR	(229)
Smad7	CD4^+^ T cells	↑	Activation of TGF-β/Smad3-IL-6 and NF-κB pathways and leads to synovial inflammation in RA	RA DMARD treated vs HC	n/a	N = 57 RA/35 HC; prospective study, flow cytometry analysis; longitudinal analysis	(230)
TNF-α	PBMC	↓	Induction of inflammation through cytokine production and a suppressive effect on Treg lymphocytes	IACON: RA vs non-RA (reactive arthritis, UA, PsA) RADAR: RA vs non-RA (UA, PsA)	IACON: 0.741 RADAR: 0.840 RA ACPA (-)/HC: 0.954	IACON: N = 64 RA/63 non-RA (11 reactive arthritis, 36 UA, 16 PsA) RADAR: N = 126 RA/31 non-RA (4 PsA, 27 UA) prospective study, no difference in the distribution of methylation levels in both RA cohorts; quantitative methylation-specific qPCR	(55)
CDKN2A	PBMC	↓	Activation of immune cells (induction of pro-inflammatory factor production, regulation of dysfunctional T and B lymphocytes)	RA vs HC	0.705	N = 75 RA/75 HC; prospective study, no data on treatment and stage of disease; methylation quantification endonuclease-resistant DNA	(66)
CXCR5	Whole blood	↑	Stimulation of excessive autoantibody production, induction of inflammatory processes	RA vs HC RA vs PsA RA vs AS RA vs SLE	RA vs HC: 0.658 RA vs AS: 0.662 RA ACPA (-) vs AS: 0.967	N= 164 RA/30 SLE/30 AS/30 PsA/30 HC; prospective study, no data on treatments and stage of diseases; targeted methylation sequencing	(231)
SMAD3	Whole blood	↓	Regulator of TGF-β signaling; supports EMT and FLS migration; potential biomarker RA	RA vs OA RA vs HC	RA vs HC: logistic regression results for various CG sites AUC for high-level clinical indicators range of 0.64-0.78 AUC for low-level clinical indicators range of; 0.63-0.72	N = 241 RA/30 OA/30 HC; prospective study, only female patients; RA patients with no data on treatment and stage of disease; unequal statistical power	(71)
CD248		↑	FLS marker; involved in fibroblast migration and cartilage erosion; expressed in early RA; potential therapeutic target				
LYST		↓	Regulates intracellular protein transport; influences cytokine production				
PRDM16		↑	Potential therapeutic target; associated with cartilage and bone regeneration				
YAP1		↓	Regulator of cell survival; affects CTGF and FLS migration; influences FLS proliferation and survival in RA				
WNT7A		↑	Regulates osteoblast differentiation and inflammatory responses; may promote FLS proliferation and modulate IL-6, IL-10, and IL-12				
C14orf180		↑	Unknown function; potential research direction in RA				
HIPK3	Whole blood	↓	regulation of cellular biological functions (proliferation, apoptosis, signal transduction)	RA vs HC RA vs AS, RA vs GOUT RA vs PsA	RA vs HC: 0.742 RA RF(+) ACPA (-) vs HC: 0.836 RA RF (-) ACPA (-) vs HC: 0.612 RA vs AS: 0.788 RA vs GOUT: 0.643 RA vs PSA: 0.696	N = 164 RA/30 AS, 30 GOUT/30 PsA; prospective study, no data on treatment and stage of disease; targeted region methylation sequencing	(62)
PCDH17	Whole blood	↓	potential clinical applications for predicting the degree of inflammation in RA patients	RA vs HC, RA vs AS, RA vs SS	RA RF(-) ACPA(-) vs HC: 0.650	N = 166 RA/24 SS/no data on SS group size; prospective study, no data on treatment and stage of disease; PCR method	(232)
HTR2A	Whole blood	↑	induction of proinflammatory cytokine production in T lymphocytes and monocytes	RA vs HC	RA vs HC: 0.757 RA RF(-) ACPA(-) vs HC: 0.966 RA RF(+) ACPA(+) vs HC: 0.846 RA RF(-) vs HC: 0.932	N = 407 RA/60 HC; prospective study, no data on treatment and stage of disease; targeted DNA methylation	(68)
ND5	Whole blood	↓	Potential influence mitochondrial function by affecting ROS production, thereby contributing to the pathogenesis of RA	RA vs HC	n/a	N = 32 RA/32 HC; prospective study, no data on treatment and stage of disease; mtDNA whole genome sequencing	(233)
RNR2		↓					
ND2		↓					

*The symbol “↑” denotes an increase (up-regulation) in DNA methylation levels, whereas the symbol “↓” indicates a decrease (down-regulation) in DNA methylation at the respective genomic regions or loci.*

AUC, Area Under the Curve; CD4+ T cells, Cluster of Differentiation 4 positive T lymphocytes; CXCR5, C-X-C Motif Chemokine Receptor 5; DMARD, Disease-Modifying Anti-Rheumatic Drugs; FLS, Fibroblast-Like Synoviocytes; HC, Healthy Controls; IACON, Inception Cohort; IL-6/IL-17, Interleukin-6/Interleukin-17; mtDNA, Mitochondrial DNA; ND2/ND5, NADH Dehydrogenase subunit 2/5; PBMC, Peripheral Blood Mononuclear Cells; PsA, Psoriatic Arthritis; qPCR/qRT-PCR, quantitative (Reverse Transcription) Polymerase Chain Reaction; RA, Rheumatoid Arthritis; RF, Rheumatoid Factor; ROS, Reactive Oxygen Species; SS, Sjögren’s Syndrome; TNF-α, Tumor Necrosis Factor alpha; UA, Undifferentiated Arthritis; ACPA, Anti-Citrullinated Protein Antibody; OA, Osteoarthritis; AS, Ankylosing Spondylitis; n/a, not assessed.

### 2.5 Quality appraisal and synthesis approach

**Figure 1:**
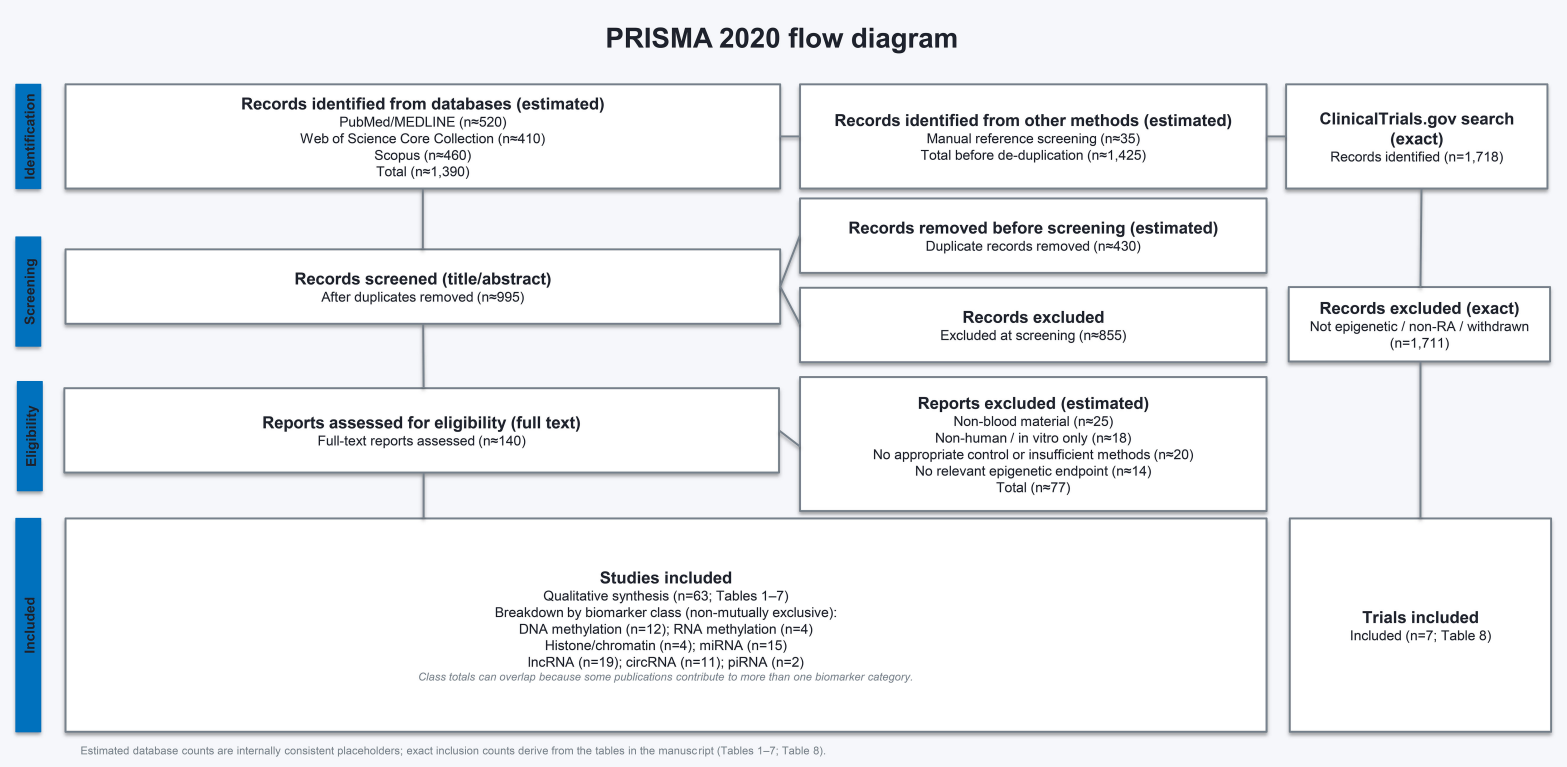
PRISMA 2020 flow diagram of literature search and study selection. Identification and screening counts could not be fully reconstructed retrospectively from database export logs and are therefore reported as not available. The diagram reports the final number of studies included in the qualitative synthesis (n = 63; **Tables 1**–**7**) and clinical trials identified through ClinicalTrials.gov (n = 7; **Table 8**). The review represents a structured narrative synthesis without quantitative meta-analysis.

The study selection process is summarized in the PRISMA 2020 flow diagram (**Figure 1**). In total, 63 publications were included in the qualitative synthesis of blood-based epigenetic biomarkers (**Tables 1**–**7**), and 7 clinical trials were identified through the registry search (**Table 8**). Because this work constitutes a structured narrative review rather than a fully systematic review with meta-analysis, exact record-level counts for database identification and screening could not be fully reconstructed retrospectively. Accordingly, the values presented in the PRISMA diagram were retrospectively estimated and reported as approximate, in order to maintain transparency of the literature selection process. No formal standardized risk-of-bias assessment tool was applied. Instead, included studies were qualitatively appraised based on study design, cohort size, methodological rigor, validation strategy, and consistency of reported findings. Accordingly, this review should be interpreted as a structured narrative synthesis rather than a formal systematic review, with the PRISMA 2020 framework applied solely to enhance transparency and reproducibility of the study selection process.

**Table 2:** A literature review on RNA methylation changes involved in RA, investigated in blood and its fractions.

Studied changes	Cell/tissue type	Direction of methylation change	Biological function/effect	Clinical comparison	ROC, AUC	Sample size/key limitations	Reference
METTL3	PBMC	↑	Induces inflammation, proliferation and aggression of FLS, as well as joint destruction	RA new active patients not treated with DMARD and/or steroids	n/a	n = 47 RA; prospective study; qRT-PCR	(75)
m6A	Whole blood	↑	Activates and enhances the aggression of FLS, inducing the synthesis of pro-inflammatory factors and oxidative stress mediators (differences are due to different cohorts/disease activity states)	new-onset RA patients not treated with corticosteroids or DMARD, 9 patients where treated with corticosteroids and immunosuppressive drugs for at least 15 days vs control group	n/a	n= 79 RA/61 Control group; prospective study; qRT-PCR	(72)
	PBMC	↓		RA vs HC	n/a	n=168 RA/59 HC qRT-PCR, MeRIP sequencing	(74)
ALKBH5, FTO	Whole blood	↓	removes mRNA methylation and affects the stability/translation of these molecules	new-onset RA patients not treated with corticosteroids or DMARD, 9 patients where treated with corticosteroids and immunosuppressive drugs for at least 15 days vs control group	n/a	n = 79 RA/61 Control group; prospective study; qRT-PCR	(72)
YTHDF2	Whole blood	↓	recognizes m6A and often directs them toward degradation or modifies their stability/translation	new-onset RA patients not treated with corticosteroids or DMARD, 9 patients where treated with corticosteroids and immunosuppressive drugs for at least 15 days vs HC	n/a	n = 79 RA/61 HC; prospective study; qRT-PCR	(72)
	PBMC			new-onset RA patients not treated with corticosteroids or DMARD vs HC	n/a	n = 74 RA/63 HC; prospective study; longitudinal analysis; qRT-PCR	(73)
METTL14	PBMC	↓	Decreased METTL14 expression is associated with reduced m6A levels and increased inflammatory activity.	RA vs HC	n/a	n=168 RA/59 HC qRT-PCR, MeRIP sequencing	(74)

*The symbol “↑” indicates an elevated level (up-regulation) of RNA methylation, while the symbol “↓” represents a reduced level (down-regulation) of RNA methylation in the corresponding transcripts*.

ALKBH5, AlkB Homolog 5 RNA Demethylase, demetylaza m6A RNA; DAS28, Disease Activity Score using 28 joints; DMARD, Disease-Modifying Anti-Rheumatic Drugs; FLS, Fibroblast-Like Synoviocytes; HC, Healthy Controls; IL-6/IL-17, Interleukin-6/Interleukin-17; m6A, N6-methyladenosine; METTL3/METTL14, Methyltransferase-Like 3/14, m6A „writers”; PBMC, Peripheral Blood Mononuclear Cells; qRT-PCR, quantitative Reverse Transcription PCR; ROS, Reactive Oxygen Species; TNFAIP3, Tumor Necrosis Factor Alpha-Induced Protein 3; YTHDF2, YTH N6-Methyladenosine RNA Binding Protein 2; n/a, not assessed.

**Table 3:** Literature review of histone modifications involved in RA analysed in blood and blood-based fractions.

Type of modification	Cell/tissue type	Direction of change	Biological function/effect	Clinical comparison	ROC, AUC	Sample size/limitations	Reference
Histone H3 acetylation (H3Ac)	PBMCs	↑ H3, ↑ HAT, ↓ HDAC3 Class I HDAC expression (HDAC1/2/3/8) ↓	Increased expression of proinflammatory genes; activation of T lymphocytes	RA vs healthy controls	n/a	n = 48 RA/48 HC; RT-qPCR; cross-sectional; potential confounding by medication, age, and sex; no longitudinal follow-up	(84)
Histone H3/H4 acetylation	Monocytes	↑ Chromatin accessibility	Amplifies systemic inflammatory response	RA vs OA vs healthy controls	n/a	n = 26 RA/23 OA/14 HC; ATAC-seq; cross-sectional; no single-cell resolution; complex *in vivo* context	(86)
Epigenetic modifications (acetylation/methylation)	T cells (Treg/Th17)	Disturbed Treg/Th17 balance, increased inflammatory response	Dysregulated acetylation and methylation	RA vs healthy controls; RA baseline vs post-treatment (methotrexate, sarilumab, JAK inhibitors)	n/a	n = 45 RA baseline + 16 RA post-treatment/17 HC; prospective design; flow cytometry; heterogeneous treatment regimens; limited stratification by therapy	(87)
Histone H3 acetylation (H3Ac)	PBMCs	Insulin-modulated acetylation	Regulates immune activation and glucose metabolism	RA patient cells; experimental modulation	n/a	Preprint study; no formally defined cohort size; preliminary results; full peer-review pending	(88)

*“↑” indicates increased (up-regulated) and “↓” indicates decreased (down-regulated) levels of epigenetic modifications or enzyme expression.*

ATAC-seq, Assay for Transposase-Accessible Chromatin using sequencing; HC, Healthy Controls; H3Ac, Histone H3 acetylation; HDAC, Histone Deacetylase; HAT, Histone Acetyltransferase; JAK, Janus Kinase; OA, Osteoarthritis; PBMCs, Peripheral Blood Mononuclear Cells; RA, Rheumatoid Arthritis; RT-qPCR, Reverse Transcription quantitative Polymerase Chain Reaction; Th17, T helper 17 cells; Treg, Regulatory T cells; n/a, not assessed.

**Table 4:** Literature review of miRNAs involved in RA analysed in blood and blood-based fractions.

miRNA	Cell/tissue type	Expression	Biological function/effect	Clinical comparison	ROC, AUC	Sample size/limitations	Reference
miR-17 miR-106b	Plasma	↓	Proposed biomarkers of RA disease activity/severity	RA vs healthy controls; high activity vs remission	miR-17: AUC = 0.74, Sensitivity = 70%, Specificity = 68% miR1-06b: AUC = 0.71, Sensitivity = 65%, Specificity = 66%	n = 50 RA (29 high activity, 21 remission)/24 HC; cross-sectional; RTqPCR; miR-17 high activity RA not different from HC; miR-106b no difference between high activity RA vs HC	(102)
miR-223	Serum	↑	Diagnostic biomarker candidate; predictor of RA risk (reported discriminator vs controls)	RA patients vs healthy controls	AUC = 0.85; Sensitivity = 80%; Specificity = 95.38%	n = 120 RA/130 HC; cross-sectional; qRT-PCR; correlational; limited control for treatment effects typical for serum biomarker studies	(104)
miR-126-3p let-7d-5p miR-431-3p miR-221-3p miR-24-3p miR-130a-3p miR-339-5p let-7i-5p	Serum	↑	Modulation of immune/inflammatory responses; potential biomarkers of RA activity and treatment response.	RA patients vs healthy controls; at risk vs HC; changes pre/post MTX	let-7d-5p AUC = 0.8360; Panel miR-221-3p, miR-24-3p, miR-130a-3p AUC = 0.8148; Panel miR-130a-3p, miR-339-5p, let-7i-5p, miR-126-3p, miR-431-3p AUC not reported	n = 50 RA/20 HC/10 at-risk; 18 RA post-MTX; cross-sectional with follow-up subset; qRT-PCR	(110)
miR-17-5p		↓					
					miR-175p AUC = 0.74;		
miR-22-3p miR-24-3p miR-140-3p miR-627-5p miR-96-5p miR-134-5p	Plasma	↑	Bioinformatically identified panel differentiating RA patients from controls; may serve as an autoimmune signature independent of active disease or seropositivity	RA vs healthy controls; validated in separate RA cohort and SLE comparison	Discovery: AUC = 0.81; Validation: AUC = 0.71; Sensitivity/Specificity: not reported	Discovery n = 167 RA/91 HC; Validation n = 32 RA/32 HC; SLE n = 12; cross-sectional; small RNA-seq (discovery) + qRT-PCR (panel validation); limited disease specificity vs SLE	(113)
miR-204-5p	Plasma exosomes	↓	Mediates communication between immune cells and synovial fibroblasts; potential diagnostic and therapeutic biomarker	RA patients vs healthy controls (discovery, replication, validation cohorts)	n/a	Discovery: pooled 9 RA/9 HC; Replication: 30 RA/30 HC; Validation: 56 RA/60 HC; cross-sectional; exosome isolation + qRT-PCR (validation); multi-stage design	(116)
miR-19b-3p miR-503-5p miR-589-5p miR-3614-5p	Serum	↑	Biomarkers for progression and response to JAK inhibitor (baricitinib) miR-19b-3p shows strongest predictive value; others exploratory	RA (early RA and advanced RA) vs healthy controls; before vs after baricitinib	AUC = 0.85 Sensitivity/Specificity: not reported	n= 44 RA/37 HC (+ comparator groups reported in study); follow-up after baricitinib (3 months); qRT-PCR profiling; heterogeneous clinical context; specificity vs other inflammatory arthritides may be limited	(114)
miR-29c-5p miR-374a-5p		↓					
miR-21-5p miR-142-3p	Serum	↑	Positively correlates with TNF-α, CRP, and ACPA; potential therapeutic targets; useful for disease activity monitoring and treatment response	Positive correlation with TNF-α, CRP, ACPA; candidate for activity monitoring/response tracking	n/a	n = 60 RA/30 HC; cross-sectional; qRT-PCR; correlational; subgroup differences may vary; treatment confounding not fully excluded	(109)
798 miRs (miRNA panel enriched in MTX responders), including miR-212-3p, miR-338-5p, miR-410-3p and miR-537	Serum, Extracellular vesicles	↑	Overexpressed in MTX responders; potential role in regulating pathogenic potential of synovial fibroblasts	Early untreated RA vs non-inflammatory controls (NICs) and PMR; changes after 6 months MTX in responders vs non-responders	n/a	n = 46 early RA/23 PMR/12 NIC; longitudinal pre/post MTX; EV isolation + broad miR profiling (reported 798 miRs) with follow-up validation in study framework; exploratory-needs independent validation	(111)
miR-186	whole blood	↓	Potential marker of disease activity in RA	RA vs HC; active vs inactive disease; ACPA- vs HC	n/a	n = 46 RA/20 HC; cross-sectional; qRT-PCR; limited external validation	(105)
miR-548ah-3p miR-378g miR-27a-5p miR-30c-2-3p	PBMC, exomiRNA	↑	Noninvasive monitoring of response to tofacitinib in MTX-resistant RA	RA (MTX resistant) vs healthy controls; before/after tofacitinib therapy	miR-548ah-3p AUC = 0.97; Sensitivity = 84.85%; Specificity = 96.15% miR-378g AUC = 0.74 miR-27a-5p AUC = 0.92; Sensitivity = 90.62%; Specificity = 84.37% miR-30c-2-3p AUC = 0.80	n = 74 RA/35 HC; longitudinal treatment monitoring; exosome isolation + qRT-PCR	(234)
miR-155	Serum	↑	Serum miR-155 correlates with disease severity; proposed together with TNF-α and TGF-β1 as prognostic markers; may reduce need for synovial sampling	RA vs healthy controls	AUC = 0.972; sensitivity 94.4%; specificity 88.9%	n = 80 RA; 40 healthy controls; cross-sectional; qRT-PCR; needs independent validation	(103)
miR-99b-5p miR-101-3p miR-431-5p	Whole blood	↑	Biomarkers for early RA detection; potential to improve patient management	RA vs healthy controls	miR-99b-5p AUC = 0.873; sensitivity 78%; specificity 94% miR-101-3p AUC = 0.873; sensitivity 78%; specificity 94% miR-431-5p AUC = 0.856; =sensitivity 73%; specificity 89%	n = 60 RA/20 HC; cross-sectional; qRT-PCR; no longitudinal validation; treatment strata explored in paper	(106)
miR-210-3p	Plasma	↓	Hypoxia-responsive miRNA; associated with immune dysregulation and inflammatory signaling; proposed diagnostic biomarker in RA	RA vs healthy controls	AUC = 0.75; Sensitivity = 66%; Specificity = 71%	n = 40 RA/40 HC; cross-sectional; qRT-PCR (plasma miRNA quantification); normalization to endogenous control; no longitudinal follow-up; treatment status not fully stratified; moderate diagnostic performance	(115)
miR-21	Serum	↓	Associated with RA context in a combined ncRNA/cytokine model; biomarker candidate	RA vs healthy controls;	n/a	n = 100 RA/100 HC; cross-sectional; qRT-PCR (serum expression analysis)	(108)
miR-7 (linked to ciRS-7 axis)	PBMC	↓	ciRS-7/miR-7 axis proposed for RA diagnosis; preliminary	RA vs healthy controls	n/a	n = 18 RA/14 HC; qRT-PCR; small sample size; preliminary	(112)

*The symbol “↑” signifies an up-regulation or increased expression of the respective miRNA, whereas the symbol “↓” denotes a down-regulation or decreased expression level of the miRNA.*

ACPA, Anti-Citrullinated Protein Antibody; EV, Extracellular Vesicles; HC, Healthy Controls; JAK, Janus Kinase; MTX, Methotrexate; PBMC, Peripheral Blood Mononuclear Cells; qRT-PCR, quantitative Reverse Transcription Polymerase Chain Reaction; RA, Rheumatoid Arthritis; SLE, Systemic Lupus Erythematosus; TNF-α, Tumor Necrosis Factor Alpha; n/a, not assessed.

**Table 5:** Literature review of lncRNAs involved in RA, investigated in blood and its fractions.

lncRNA	Cell/tissue type	Expression	Biological function/effect	Clinical comparison	ROC, AUC	Sample size/limitations	Reference
MEG3	PBMC (cell-intrinsic expression)	↓	Suppression of inflammation through NF-κB regulation; expression-based biomarker	RA vs healthy controls; correlation with joint count and disease activity	n/a	n = 82 RA/15 HC + 24 RA synovial fluid/10 OA; cross-sectional; qRT-PCR	(125)
NEAT1	PBMC	↑	Macrophage activation and production of pro-inflammatory cytokines; functional contribution to RA pathogenesis	Functional relevance demonstrated *in vitro* and *in vivo*; differential expression	n/a	Human PBMC/exosome studies + mouse model; qRT-PCR	(126)
IFNG-AS1	PBMC	↑	Diagnostic and/or therapeutic biomarker candidate	RA vs healthy controls; expression correlates with disease activity	n/a	n = 50 RA/30 HC; cross-sectional; qRT-PCR; ROC utility suggested but no numeric AUC reported	(127)
PVT1	Serum	↑	Diagnostic and/or therapeutic markers	RA vs healthy controls (and OA comparison)	AUC = 0.836; Sensitivity = 82.5%; Specificity = 100%	n = 40 RA/40 OA/40 HC; cross-sectional; qRT-PCR	(129)
SNHG14	PBMC	↑	RA disease biomarker; correlates with disease activity via MINK1-JNK pathway activation	RA vs healthy controls	n/a	n = 40 RA/40 HC cross-sectional; qRT-PCR;	(143)
THRIL HOTAIR	Serum	↑	Diagnostic lncRNAs associated with RA activity through target gene regulation	RA vs healthy controls; remission vs active RA	THRIL AUC= 0.73 Sensitivity =73.08% Specificity =100% HOTAIR AUC = 0.69 Sensitivity =61.58% Specificity = 100%	n = 52 RA (23 remission/29 active)/56 HC; cross-sectional; qRT-PCR	(130)
GAPLINC	Serum	↑	RA activity marker	RA vs healthy controls	n/a	cohort size and methods not reported- abstract only	(137)
Linc00152 lnc-ADM-1	PBMC	↑	Potential RA biomarker, potentially involved in its pathogenesis	RA vs healthy controls	panel AUC = 0.886	n=155 RA/145 HC/59 SLE/59 pSS cross-sectional; qRT-PCR;	(128)
ITSN1-2 lnc-FTH1-7	Whole blood	↓	An innovative diagnostic model based on PTPRE, neutrophil count, and RDW, which may serve as a potential tool for diagnosing seronegative RA patients	seronegative RA vs healthy controls	AUC refers to composite model (PTPRE + neutrophils + RDW), not individual lncRNAs AUC = 0.6709	n = 96 RA/40 HC; cross-sectional; qRT-PCR + clinical parameters	(135)
ADGRE5 FAM157A PTPN6 PTPRE	Whole blood	↑	An innovative diagnostic model based on PTPRE, neutrophil count, and RDW, which may serve as a potential tool for diagnosing seronegative RA patients	seronegative RA (SNRA) vs healthy controls	PTPRE AUC = 0.6709	n=96 RA/40 HC; cross-sectional; qRT-PCR	
SNHG3	Serum	↓	A potential diagnostic biomarker for RA, involved in the regulation of inflammatory responses and oxidative stress through negative modulation of miR-128-3p	RA vs healthy controls	AUC data not reported- abstract only, sensitivity =87.5%, specificity= 84.4%	abstract only; cohort size not reported	(138)
ITGB2-AS1	Serum	↑	Predictive diagnostic biomarker (combined with ICAM-1)	RA vs healthy controls; OA comparison	AUC=0.772, Sensitivity=54.84%, Specificity=100%	n=43 RA/22 HC/35 OA; cross-sectional; qRT-PCR	(131)
NORAD	Serum	↑	RA progression mediator via sponging miR-204-5p; regulation of clinical indicators	RA vs healthy controls	AUC = 0.91; Sensitivity =80.5%; Specificity =88.5%	n = 77 RA/52 HC; cross-sectional; qRT-PCR	(132)
TCONS_I2_00013502, ENST00000363624	Serum (exosomes)	↑	Exosomal lncRNAs improving RA diagnosis when combined with ACPA	RA vs healthy controls	TCONS_I2_00013502 AUC = 0.870; Sensitivity =93%; Specificity =68.7% ENST00000363624 AUC = 0.864; Sensitivity =81.3%; Specificity =78.1%	Validation cohort: n=32 RA/32 HC; exploratory; qRT-PCR small sample size; exploratory design	(136)
NEAT1	Serum	↑	Circulating biomarker correlating with RA activity	RA vs healthy controls	n/a	n=100 RA/100 HC; cross-sectional; qRT-PCR	(108)
OIP5-AS1 (panel of 64 lncRNAs)	PBMC	↑	Potential biomarkers in RA. PBMC-based diagnostic lncRNA panel with ROC-defined performance.	RA vs healthy controls	OIP5-AS1 AUC = 0.915 Combined panel (LINC00494 + TSP0AP1-AS1 + MCM3AP-AS1 + LINC01588 + OIP5-AS1) AUC = 0.920 The remaining lncRNAs in the panel have approximate AUCs reported in the article ranging from 0.654 to 0.915	Screening: n=3 RA/3 HC; Validation: n=38 RA/36 HC; qRT-PCR panel	(134)
50 lncRNA, including LINC00494, TSP0AP1-AS1, MCM3AP-AS1 LINC01588	PBMC	↓	Broad transcriptional deregulation associated with RA. Global lncRNA expression changes without diagnostic ROC evaluation.	RA vs healthy controls	n/a	Discovery and validation cohorts; qRT-PCR	(136)
NUTM2A-AS1	Whole blood	↑	A potential mechanism of inflammatory modulation via diet, involving NUTM2A-AS1 and CCR3	Pre- vs post-intervention in active RA	n/a	Discovery phase: n=7; Validation phase: n=21; single arm pilot; qRT-PCR	(145)
RNA143598	Serum	↑	Diagnostic biomarker candidate	RA vs healthy controls	AUC = 0.77	n= 39 RA/53 HC cross sectional; qRT-PCR; further validation needed	(144)
MEG3 (expression + genetics)	PBMC	↓	Expression and genetic polymorphisms correlate with RA severity; functional validation in FLS.	RA vs healthy controls	n/a	n = 551 RA/595 HC; SNP genotyping + qRT-PCR; *in vitro* functional assays	(124)
KCNQ1OT1	Serum	↓	Disease activity biomarker; enhanced diagnostic power in combination with miR-210	RA vs healthy controls	Combined miR-210 + KCNQ1OT1: AUC = 0.949, Sensitivity = 83.58%, Specificity = 93.33%	n=67 RA/60 HC; cross-sectional; qRT-PCR	(133)

*The symbol “↑” signifies an up-regulation or increased expression of the respective lncRNA, whereas the symbol “↓” denotes a down-regulation or decreased expression level of the lncRNA.*

ACPA, Anti-Citrullinated Protein Antibody; FLS, Fibroblast-Like Synoviocytes; HC, Healthy Controls; MTX, Methotrexate; PBMC, Peripheral Blood Mononuclear Cells; qRT-PCR, quantitative Reverse Transcription Polymerase Chain Reaction; RA, Rheumatoid Arthritis; RDW, Red Cell Distribution Width; SNP, Single Nucleotide Polymorphism; n/a, not assessed; EV, Extracellular Vesicles.

**Table 6:** Literature review of circRNAs involved in RA, investigated in blood and its fractions.

circRNA	Cell/tissue type	Expression	Biological function/effect	Clinical comparison	ROC, AUC	Sample size/limitations	Reference
circNUP214	PBMC	↑	Modulates Th17 cell response; positively correlates with IL-23R expression and Th17 frequency; mechanistically involved in RA inflammation	RA vs healthy controls	AUC = 0.76; Sensitivity = 43%; Specificity = 96%	n = 28 RA/28 HC; cross-sectional; qRT-PCR; functional correlation with Th17/IL-23R; moderate sensitivity; requires validation in larger cohorts	(160)
hsa_circ_0000175 hsa_circ_0008410	Whole blood	↑	Potential biomarkers associated with RA disease activity	RA vs HC; discrimination from SLE and AS	hsa_circ_0000175AUC = 0.835; Sensitivity = 86.21%; Specificity = 73.33% hsa_circ_0008410 AUC = 0.804; Sensitivity = 55.17%; Specificity = 95.56% Combined model (hsa_circ_0000175 + hsa_circ_0008410) AUC = 0.971; Sensitivity = 93.10%; Specificity = 93.33%	Validation cohort: n=63 RA/21 HC (total n=87 RA/45 HC; cross-sectional; qRT-PCR	(161)
hsa_circ_0001200; hsa_circ_0001566; hsa_circ_0003972 hsa_circ_0008360	PBMC	↑	Candidate diagnostic circRNAs in RA	RA vs healthy controls	n/a	Discovery by RNA-seq (n = 3 RA/3 HC); validation by RT-qPCR (n = 10 RA/10 HC); small sample size; exploratory	(164)
hsa_circ_0140271	PBMC	↑	Potential diagnostic circRNAs with sex-specific relevance	Female RA **vs** female healthy controls; female OA; female AS	RA vs HC: AUC = 0.704 Sensitivity = 41.9%, Specificity = 100% RA vs AS: AUC = 0.922 Sensitivity = 100%, Specificity =80.6% RA vs OA: AUC = 0.868 Sensitivity = 100%, Specificity = 64.5% RA vs AS + OA: AUC = 0.818 Sensitivity = 100%, Specificity = 64.5%	51 RA (female subset 31) vs 47 female healthy controls, plus 24 female OA and 7 female AS; one-center study; modest cohort	(165)
hsa_circ_0005008 hsa_circ_0005198	Plasma	↑	IDisease-activity-associated circRNAs in newly diagnosed RA	New-onset RA vs HC, SLE	hsa_circ_0005008 AUC = 0.829; Sensitivity = 95%; Specificity = 60% hsa_circ_0005198 AUC = 0.783; Sensitivity = 55%; Specificity = 90.8%;	n = 49 RA/40 HC/25 SLE; cross-sectional; qRT-PCR; early RA cohort	(166)
hsa_circ_101328	PBMC	↓	High-performance diagnostic circRNA	RA vs healthy controls	AUC = 0.957	Screening: microarray (n = 4 RA/4 HC); validation: n = 20 RA/10 SLE/20 HC; single-center; needs external validation	(167)
hsa_circ_0003353	PBMC/Serum	↑	Functional circRNA influencing inflammatory responses	RA vs healthy controls	n/a	n = 55 RA/30 HC; cross-sectional; mechanistic focus; no independent validation	(168)
hsa_circ_0005567	Plasma	↑	Biomarker of RA disease activity	RA (overall and high activity) vs healthy controls	n/a	n = 45 RA/26 HC; cross-sectional; no longitudinal analysis	(169)
hsa_circ0000175	Serum	↑	Induces pyroptosis via miR-31-5p/GSDME axis; potential therapeutic target	RA vs healthy controls	n/a	n=28 RA/28 HC in mechanistic subset; single-center; functional emphasis; not a diagnostic cohort	(163)
hsa_circ_0003914	Serum/plasma exosomes	↑	Candidate biomarker and therapeutic target	RA vs healthy controls	n/a	n=57 RA/33 HC single-center study; cross-sectional	(170)
ciRS-7	PBMC	↑	circRNA-miRNA axis (ciRS-7/miR-7) implicated in RA diagnosis	RA vs healthy controls	AUC = 0.766; Sensitivity= 77.8%; Specificity= 78.6%	n = 18 RA/14 HC; preliminary study; limited sample size	(112)

*The symbol “↑” signifies an up-regulation or increased expression of the respective circRNA, whereas the symbol “↓” denotes a down-regulation or decreased expression level of the circRNA.*

AUC, Area Under the Curve; AS, Ankylosing Spondylitis; circRNA, Circular RNA; FLS, Fibroblast-Like Synoviocytes; HC, Healthy Controls; IL-23R, Interleukin-23 Receptor; MTX, Methotrexate; PBMC, Peripheral Blood Mononuclear Cells; qRT-PCR, quantitative Reverse Transcription Polymerase Chain Reaction; RA, Rheumatoid Arthritis; SLE, Systemic Lupus Erythematosus; ROC, Receiver Operating Characteristic; EV, Extracellular Vesicles; n/a, not assessed.

**Table 7:** A literature review on piRNAs involved in RA, studied in blood and its fractions.

piRNA	Cell/tissue type	Expression	Biological function/effect	Clinical comparison	ROC AUC	Sample size/limitations	Reference
piR-hsa-27620 piR-hsa-27124	PBMC	↑	Candidate piRNAs with altered expression in RA; potential diagnostic biomarkers	RA vs healthy controls	n/a	Discovery cohort: n = 3 RA/3 HC; cross-sectional; RT-qPCR; very small sample size; exploratory	(175)
piR-hsa-27124 piR-hsa-35982	PBMC	↑	piR-hsa-35982 proposed as a biomarker for RA, including seronegative (RF-) patients	RA vs healthy controls	n/a	n = 37 RA/20 HC; cross-sectional; RT-qPCR; no ROC analysis reported	(176)
piR-has-35982	Plasma	↓	Circulating piRNA with altered expression in RA; tissue-specific regulation			Small cohort; cross-sectional; RT-qPCR; plasma vs PBMC directionality differs	

*The symbol “↑” signifies an up-regulation or increased expression of the respective piRNA, whereas the symbol “↓” denotes a down-regulation or decreased expression level of the piRNA.*

AUC, Area Under the Curve; EV, Extracellular Vesicles; HC, Healthy Controls; PBMC, Peripheral Blood Mononuclear Cells; piRNA, Piwi-interacting RNA; qRT-PCR, quantitative Reverse Transcription Polymerase Chain Reaction; RA, Rheumatoid Arthritis; RF, Rheumatoid Factor; ROC, Receiver Operating Characteristic; n/a, not assessed.

**Table 8:** Overview of clinical trials on epigenetic alterations in rheumatoid arthritis available in ClinicalTrials.gov.

NCT no.	Subject of the study	Study type	Number enrolled (actual/estimated)	Intervention/Treatment	Reference
NCT02742337	Epigenetic signatures (EGS)	Observational	30 (estimated)	Two patient groups: the test group consisted of individuals with RA, and the control group included subjects who did not meet the American College of Rheumatology’s classification criteria for RA	(235)
NCT03149796	Tocilizumab Effect on microRNA Expression	Observational	120 (actual)	Two patient groups: 60 RA patients treated with tocilizumab, according to the local clinical guidelines and 60 healthy controls	(196)
NCT04204603	CKD-506, a novel selective HDAC6 inhibitor on RA symptoms	Interventional	122 (estimated)	Evaluation of the efficacy, safety, pharmacokinetics, and biomarker effects of CKD-506 in adult patients with moderate to severe rheumatoid arthritis who have shown inadequate response to methotrexate, conducted in parallel groups under a double-blind design	(204)
NCT02350491	Changes in miRNA expression patterns	Observational	50 (actual)	Three patient groups: Pregnant women suffering from Rheumatoid Arthritis, Pregnant women suffering from Systemic Lupus Erythematosus and Healthy pregnant woman	(236)
NCT05914337	Changes in miRNA expression	Interventional	60 (actual)	Two patient groups: 30 rheumatoid arthritis patients and 30 healthy controls	(237)
NCT06527534	The effect of Filgotinib on miRNA expression	Interventional	30 (estimated)	Two patient groups: 15 patients treated with Filgotinib. 15 patients treated with Adalimumab (used as a comparison group)	(192)
NCT05808309	Identificati n of Genomic Biomarkers including non-coding RNA	Observational	0 (actual)	Four patient groups: Patients with a diagnostic of rheumatoid arthritis with an age of onset before 60 years, from 65 years, patients with a diagnostic of osteoarthritis without RA, Relatives to PRt, with RA (with any age of onset) or without RA	(206)

HDAC, Histone Deacetylase; miRNA, MicroRNA; NCT, ClinicalTrials.gov Identifier; RA, Rheumatoid Arthritis; CKD, compound/drug code; qRT-PCR, quantitative Reverse Transcription Polymerase Chain Reaction; EGS, Epigenetic Signatures; actual/estimated, actual or estimated number of enrolled participants; n/a, not assessed; PRt, first-degree relative; HC, Healthy Controls.

## 3 Epigenetic mechanisms in rheumatoid arthritis

Epigenetics is a field that investigates changes in gene expression that occur through chromosomal modifications rather than alterations in the DNA sequence itself (22). The main mechanisms include DNA methylation, histone modifications, and gene regulation by non-coding RNAs. The epigenome is highly influenced by environmental factors- aging, diet, and stress can induce epigenetic alterations that contribute to disease development (23, 24). These environmentally induced epigenetic modifications are increasingly recognized as key contributors to complex disease phenotypes. Current evidence indicates that epigenetic changes are involved in the pathogenesis of various disorders, including cancer (25), metabolic diseases such as type 2 diabetes (26), neurological disorders (27) and autoimmune diseases (28) including RA (29). Studies have demonstrated that patients with RA exhibit global DNA hypomethylation in immune cells, leading to excessive expression of pro-inflammatory genes (16). In addition, hypermethylation of anti-inflammatory genes has been observed, further sustaining chronic inflammation (30). Histone modifications also play an important role in RA by regulating genes involved in inflammatory responses, cell proliferation, and apoptosis of synovial cells (31). It is hypothesized that RA develops in genetically predisposed individuals through an interplay between genetic variability, epigenetic modifications, and environmental triggers, which may be initiated by stochastic events such as injury or infection (2, 32, 33).

DNA methylation is one of the principal epigenetic mechanisms. It primarily affects cytosine residues within CpG dinucleotides, which frequently cluster in CpG islands. In humans, this modification is catalyzed by DNA methyltransferases (DNMTs), which transfer a methyl group from S-adenosylmethionine to cytosine. Because approximately 60-70% of mammalian gene promoters contain CpG islands, methylation has a substantial impact on transcriptional regulation (34). Methylated DNA promotes heterochromatin formation, leading to nucleosome compaction and reduced accessibility for transcription factors, ultimately resulting in gene silencing (35). Conversely, active DNA demethylation removes methyl groups from cytosine residues, increases chromatin accessibility, and facilitates transcriptional activation (36). Together, these opposing processes help maintain stable gene-expression patterns, which is essential for understanding persistent inflammatory phenotypes observed in diseases such as RA.

Another form of epigenetic modification is RNA methylation, which occurs post-transcriptionally, influencing RNA splicing, translation, stability, and degradation (37). Because these modifications are reversible, they dynamically modulate the expression of mRNAs, cytokines, and regulatory genes (38). Several types of RNA methylation have been identified, including m6A, m1A, m5C, and m7G, with m6A being the most abundant and best characterized (39). m6A marks are deposited by a methyltransferase complex primarily composed of METTL3 and METTL14, together with regulatory cofactors such as WTAP. The modification is removed by demethylases FTO and ALKBH5, whereas ‘reader’ proteins, most prominently the YTH domain family that bind to m6A-modified transcripts and determine their fate (40–42). Through these coordinated activities, RNA methylation fine-tunes gene-expression programs relevant to immune activation and chronic inflammation.

Histone modifications constitute another major layer of epigenetic regulation. Histones, which package DNA into nucleosomes, possess N-terminal tails that undergo multiple enzyme-driven post-translational modifications, including acetylation, methylation, phosphorylation, and ubiquitination (43). Among these, acetylation and methylation are the best characterized. Histone acetylation involves the addition of acetyl groups to lysine residues, reducing histone-DNA affinity, relaxing chromatin structure, and promoting transcriptional activation. This modification is highly dynamic and is controlled by the opposing activities of histone acetyltransferases (HATs) and histone deacetylases (HDACs) (44). Histone methylation, catalyzed by histone methyltransferases (HMTs), can either activate or repress gene expression depending on the specific residue and degree of methylation (45). Phosphorylation of histones also influences chromatin dynamics, although its biological roles are less well characterized. Histone ubiquitination, involving the attachment of ubiquitin to lysine residues, is most commonly linked to transcriptional repression, although context-dependent activating functions have also been described (46).

Non-coding RNAs represent another major epigenetic regulatory mechanism. ncRNAs encompass a broad group of transcripts with diverse roles, some of which participate in epigenetic regulation. Based on their length, ncRNAs can be classified into diverse groups - from short species such as miRNAs (micro RNA), siRNAs and piRNAs to longer forms like lncRNAs (long non-coding RNAs) and circRNAs (circular RNAs) - some of which exert regulatory influence on epigenetic processes including DNA methylation, chromatin remodeling and mRNA stability (47). Accumulating evidence demonstrates that ncRNAs regulate key epigenetic processes, including DNA methylation, histone modifications, and sequence-specific control of mRNA translation and stability (48–50). In rheumatoid arthritis, ncRNAs function as both regulators and effectors of inflammatory signaling, shaping the activity of lymphocytes, macrophages, and synovial fibroblasts and thereby contributing to chronic synovial inflammation (51).

The following subsections summarize the best-characterized classes of ncRNAs, including miRNAs, lncRNAs and circRNAs, and introduce additional RNA species that have received comparatively little attention to date, such as siRNAs, piRNAs, Y RNAs (Ro60-Y RNA complexes), snRNAs, snoRNAs and tRNAs. Expanding the scope beyond the traditionally studied ncRNA groups provides a more complete understanding of RNA-mediated epigenetic regulation and highlights regulatory mechanisms that may have been overlooked in rheumatoid arthritis research. By integrating emerging evidence on these lesser-known ncRNA classes, this review aims to open new research avenues, clarify their potential contribution to RA pathogenesis, and support the identification of novel diagnostic biomarkers and therapeutic targets.

## 4 DNA methylation in rheumatoid arthritis

DNA methylation in RA has been studied for several decades, whereas RNA methylation has only recently emerged as an additional epigenetic layer. However, the majority of current evidence still focuses predominantly on DNA methylation abnormalities. Early on, researchers observed global hypomethylation, particularly in rheumatoid arthritis synovial fibroblasts (RASFs), although the extent of this phenomenon in peripheral immune cells remains more heterogeneous across studies (52). Additionally, it has been shown that these changes lead to altered expression of genes regulating DNA methylation dynamics, including DNMTs and TET enzymes that intensify the pathogenesis of the disease (16, 53). In the course of RA, hypermethylation and decreased expression of anti-inflammatory genes also occur, which further sustains and amplifies inflammation (54). Various studies have been conducted to identify diagnostically and therapeutically useful biomarkers of RA, particularly in patients who do not produce RF or ACPA. Subsequently, selected biomarkers are discussed, which, according to the literature, may have potential diagnostic or therapeutic significance.

Pitaksalee et al. developed qMSP-based assays to identify blood-derived epigenetic markers for RA, identifying TNF-α methylation as the most promising candidate (55). The researchers compared methylation levels in peripheral blood mononuclear cells from RA patients (n = 64, IACON database; n = 126, RADAR database and from patients with other typical joint types: reactive, undifferentiated and psoriatic arthritis (PsA) (n = 63, IACON; n = 31, RADAR). TNF-α plays a fundamental role in RA pathogenesis, driving synovial inflammation, angiogenesis and immune cell activation (56, 57). Hypomethylation of the TNF-α gene has been observed in the course of RA. These changes have been shown to significantly distinguish patients progressing to RA from those with other forms of arthritis. Moreover, no correlations were found between TNF-α values and clinical or demographic variables, which grants this biomarker independent value as an indicator for RA detection. A DNA methylation threshold of 4.5% was established to classify patients into low- and high-risk groups for progression to RA. Based on this, the odds ratio in the high-risk group from the IACON database was 8.4, with a sensitivity of 68.7% and specificity of 79.4%. The positive predictive value (PPV) was 77.2%, the negative predictive value (NPV) 71.4%, and AUC of 0.741. Even better results were obtained for patients from the RADAR database, where the odds ratio for developing RA in the high-risk group was 27.6, with a sensitivity of 84.1%, specificity of 83.8%, PPV of 95.4%, NPV of 56.5%, and AUC of 0.840. The differences between the two groups are due to a revised patient classification system implemented after 2015, which was based solely on the 2010 EULAR classification criteria and excluded patients whose symptoms resolved within 1–3 months (55). Although these results are promising, they require validation in independent, prospectively collected cohorts before TNF-α methylation can be established as a reliable diagnostic biomarker. These findings indicate that assessment of DNA methylation changes in TNF-α has high diagnostic value. Moreover, adding this biomarker to the currently used classification criteria increased diagnostic accuracy from 0.917 to 0.954, and among ACPA (–)patients from 0.892 to 0.954. The introduction of the additional TNF-qMSP test appears to have significant added value and potential as a biomarker, particularly in the diagnosis of seronegative patients with early RA. Another biomarker that has recently attracted researchers’ attention is the CXC chemokine receptor 5 (CXCR5). A strong association of this surface protein with the pathogenesis of autoimmune diseases, including RA, has been observed. CXCR5 contributes to lymphocyte trafficking and ectopic lymphoid organization, processes highly relevant to RA pathogenesis (58). CXCR5 is upregulated in RA synovial tissue and expressed on multiple infiltrating immune cell types, including B cells, T cells, macrophages, and endothelial cells (59) In contrast, peripheral blood CXCR5^+^ B cells are decreased in RA patients compared with healthy controls, suggesting altered trafficking or receptor regulation during chronic inflammation (60). These findings likely reflect the migration of CXCR5^+^ B cells toward the inflamed synovium, rather than an overall decrease in CXCR5 expression. Significant methylation changes have also been identified within this gene specifically at site cg04537602 between patients with RA, osteoarthritis, and healthy individuals.

Moreover, methylation levels at this site correlate with the severity of inflammation in RA patients (61). Based on these findings, Shi et al. compared methylation levels at site cg19599951 of the CXCR5 gene in peripheral blood from patients with RA (n = 164), SLE (n = 30), ankylosing spondylitis (AS) (n = 30), PsA (n = 30), Sjögren’s syndrome (SS) (n = 24), and healthy controls (HC) (n = 30) (61). The study revealed significant differences in methylation levels between RA and AS patients, as well as between RA patients and HC. RA patients exhibited distinctly higher methylation levels than those with AS and HC. In other groups, no statistically significant differences in CXCR5 methylation were found. As in the previously discussed study, RA patients were subdivided according to the presence of autoantibodies. The researchers then performed ROC analysis to assess the ability of CXCR5 methylation to distinguish RA subgroups from healthy controls and AS patients. The results indicated that, for the entire RA group, measuring CXCR5 methylation changes has limited diagnostic value when compared to healthy controls (AUC = 0.658) and similarly when compared to AS patients (AUC = 0.662). For individual RA subgroups compared with AS patients, diagnostic performance varied (AUC ranging from 0.624 to 0.967), with the best results obtained for ACPA (–) patients. However, these results require replication in larger and independently collected AS cohorts, as the diagnostic confidence intervals were wide and subgroup analyses may be underpowered. Thus, this method shows potential for differentiating ACPA (–) RA patients from those with AS, particularly in cases of seronegative patients presenting with atypical symptoms. However, to better assess the clinical utility of this approach, studies involving a larger cohort of AS patients are necessary. The study also lacked comparative data between RA subgroups and healthy controls. Shi et al. additionally considered clinical features and patient data such as age, sex, ESR, and CRP values. When these variables were included alongside CXCR5 methylation levels in the analysis, the diagnostic value of the method improved markedly- the AUC increased from 0.662 (for CXCR5 methylation alone) to 0.934- allowing for a clear distinction between RA and AS patient groups. For similar study groups, Jiang et al. conducted research investigating the diagnostic potential of DNA methylation levels of the HIPK3 gene (62). HIPK3 encodes a kinase involved in transcriptional regulation and cellular stress responses (63). It has been demonstrated that HIPK3 exhibits significantly lower methylation levels in the peripheral blood of RA patients, which negatively correlates with CRP levels (64). Jiang et al. analyzed differences in methylation levels across several rheumatic diseases, with the most statistically significant differences observed in patients with RA, SLE, and gout. ROC curve analysis for mean HIPK3 methylation levels yielded AUC values of 0.742, 0.701, and 0.948, respectively. Nevertheless, the inclusion of multiple heterogeneous disease groups raises concerns about disease-specificity, and future studies should validate HIPK3 methylation in strictly defined early-RA cohorts and indicated an added diagnostic value of this method for the mentioned diseases. The RA patient group was further divided into four subgroups based on the presence of autoantibodies. The best diagnostic performance (AUC = 0.836) was observed in RF(+)/ACPA (–) patients, while the lowest was found in seronegative patients (AUC = 0.612). Despite this lower value, it was still sufficient to suggest potential improvement in the diagnosis of autoantibody-negative patients. ROC analyses were also conducted to differentiate RA from other inflammatory joint diseases- AS, gout, and PsA-yielding AUC values of 0.788, 0.643, and 0.696, respectively. These findings suggest that HIPK3 methylation levels have added value in distinguishing RA from other inflammatory joint conditions.

Another biomarker tested as a potential candidate for aiding RA detection is CDKN2A. CDKN2A encodes cell-cycle regulators implicated in dysregulated immune-cell proliferation in RA (65). In RA, CDKN2A undergoes hypomethylation and increased expression, which activates immune cells by inducing the production of pro-inflammatory mediators and modulating aberrant T and B lymphocytes, thereby promoting inflammation progression (66). Consistently with the role of CDKN2A in immune dysregulation, Li et al. performed a comprehensive analysis of autophagy-related genes (ARGs) in RA and identified CDKN2A as one of five key marker genes associated with disease susceptibility. Using a generalized linear model, the authors constructed a diagnostic nomogram integrating CDKN2A together with TP53, ATG16L2, FKBP1A and GABARAPL1, which robustly discriminated RA patients from healthy individuals. The model demonstrated high predictive performance across multiple independent datasets, with AUC values exceeding 0.85 in all validation cohorts, underscoring the diagnostic potential of CDKN2A within an autophagy-based biomarker panel (67). Moreover, Gravand et al. investigated CDKN2A methylation in peripheral blood mononuclear cells as a potential diagnostic biomarker for RA (66). The study included 75 RA patients and 75 healthy controls, and ROC analysis yielded an AUC of 0.705, with 72% sensitivity and 77.3% specificity. These results indicate that CDKN2A methylation possesses moderate discriminatory power and may serve as a promising component of a multi-marker diagnostic panel rather than a standalone test. Importantly, CDKN2A methylation levels showed no significant correlations with CRP or ESR, suggesting that this epigenetic alteration reflects disease susceptibility rather than inflammatory activity, which limits its potential utility for monitoring disease activity.

The most recent and promising research on improving RA detection through DNA methylation changes was conducted by Zhao et al. (68). They analyzed DNA methylation of the HTR2A gene (serotonin receptor 5-hydroxytryptamine 2A) in peripheral blood samples from RA patients and healthy controls. Previous studies link HTR2A polymorphisms to heightened pro-inflammatory cytokine responses in RA (69). Additionally, gene-gene interactions between a protective HTR2A haplotype and the shared epitope allele of HLA-DRB1 were correlated with positive RA autoantibody status (70). Zhao et al. demonstrated significantly higher methylation levels at HTR2A cg15692052 in RA patients. Furthermore, methylation levels positively correlated with ESR and CRP levels. Incorporating HTR2A methylation into standard clinical assessments increased the AUC for distinguishing RA patients from healthy controls to 0.757. Comparable results were observed for RA subgroups stratified by autoantibody status: double-negative RF/ACPA patients achieved AUC = 0.966, double-positive RF/ACPA AUC = 0.846, and RF-negative patients AUC = 0.932. These findings highlight the potential of HTR2A methylation as a highly effective biomarker for early RA detection, particularly in seronegative patients, where diagnostic performance was the highest among all studied groups (68). However, because subgroup analyses involved smaller sample sizes, these high AUC values should be interpreted with caution. However, as the analysis was based on a single CpG site, further multi-site and multi-cohort validation is required to confirm the robustness of this biomarker. In a separate study, Zhao J. et al. expanded the investigation of methylation markers by analyzing 50 CpG sites and evaluating their diagnostic potential in relation to clinical parameters. Epigenetic markers may correlate with clinical measures of RA activity, such as the number of tender and swollen joints, CRP levels, and disease-specific autoantibodies. Zhao J. et al. investigated 50 CpG methylation sites and, using logistic regression, assessed their diagnostic potential in relation to clinical parameters (71). Parameters included swollen joint count, tender joint count, ESR, CRP, RF, ACPA, VAS, and DAS28-CRP/DAS28-ESR. Patients were categorized into high- and low-value groups for each clinical parameter, based on median, mean, or clinically relevant cutoffs. AUC values ranged from 0.64 to 0.78, sensitivity from 0.57 to 0.88, and specificity from 0.42 to 0.77. The highest diagnostic performance was observed for patients with a high number of tender joints (AUC = 0.78, sensitivity: 0.75, specificity: 0.72). The results indicated that DNA methylation levels at 7 CpG sites exhibited statistically significant correlations with the assessed clinical parameters. Given the large number of CpG sites tested, the risk of false-positive associations is non-negligible, and correction for multiple testing is essential in future analyses. Additionally, specific CpG sites showed significant correlations with clinical measures, such as: chr1:235998714 positively correlated with the number of tender joints; chr1:211500151 positively correlated with the number of swollen joints; chr15:67357339, chr3:13895991, chr14:105055171 negatively correlated with RF; chr10:70231628 positively correlated with RF; chr3:13895991 negatively correlated with ACPA. The study also assessed whether patient-specific traits such as age, weight, and height influenced methylation. Several CpG sites showed significant positive or negative correlations with age and height, indicating that some selected biomarkers are affected by non-modifiable patient factors. This highlights the need to identify CpG markers that remain stable across demographic variables. Despite this, the study demonstrates that genome-wide methylation markers can aid in identifying disease susceptibility and progression trends. Future research should aim to reduce the number of CpG sites required for analysis and identify biomarkers that are independent of immutable patient characteristics such as age or height. Overall, these results highlight the diagnostic potential of DNA methylation changes in RA, particularly in seronegative patients. A summary of the main DNA methylation biomarkers investigated in blood and its fractions is presented in **Table 1**.

## 5 RNA methylation in rheumatoid arthritis

Luo et al. conducted a study on the presence of m6A methylation in peripheral blood and the mRNA expression of METTL3, METTL14, WTAP, ALKBH5, FTO, and YTHDF2 in peripheral blood cells (72). They demonstrated a statistically significant increase in m6A levels accompanied by a decrease in the mRNA expression of ALKBH5, FTO, and YTHDF2 compared to the control group. Additionally, the reduction in FTO mRNA expression correlated with DAS28-ESR, DAS28-CRP, IgG antibody titers, and the lymphocyte-to-monocyte ratio, whereas YTHDF2 mRNA expression correlated with RBC count, the percentages of lymphocytes and neutrophils, the neutrophil-to-lymphocyte ratio, and the lymphocyte-to-monocyte ratio. No correlations were found between ALKBH5 mRNA expression and clinical symptoms of RA. Similar results for YTHDF2 protein were reported by Yao et al. (73). Notably, Luo et al. examined total RNA from whole peripheral blood, which may partly account for discrepancies with studies analyzing PBMCs rather than whole blood.

Completely different results were obtained by Tang et al., who also examined m6A levels and METTL14 in peripheral blood mononuclear cells (74). They reported a decrease in METTL14 concentration in patients with active RA compared to patients in remission and healthy controls. This decrease led to reduced m6A expression. Furthermore, m6A levels negatively correlated with DAS28. The reduced m6A expression induced the secretion of proinflammatory cytokines by PBMCs, thereby exacerbating joint inflammation. These findings contrast with Luo et al., likely reflecting differences in cell populations analyzed and disease-activity stratification. In addition, Wang et al. observed significantly increased METTL3 expression in PBMCs and monocytes compared to healthy controls (75). Given the heterogeneity of sample types, disease activity states and methodological approaches across studies, these findings should be interpreted cautiously. These studies suggest that changes in m6A levels are likely due to alterations in the concentrations of methylation-related enzymes. Taken together, the studies underscore the relevance of RNA methylation in RA pathogenesis and indicate its potential yet still preliminary diagnostic utility, pending multi-cohort validation. The key findings are summarized in **Table 2**.

## 6 Histone modifications in the pathogenesis of rheumatoid arthritis

Histone acetylation of lysine residues particularly on histones H3 and H4 has been highlighted as a relevant epigenetic mechanism in RA. Acetylation by HATs increases chromatin accessibility, whereas HDAC-mediated deacetylation promotes chromatin compaction and transcriptional repression; accordingly, the HDAC/HAT activity balance may serve as a proxy for overall transcriptional dynamics (76, 77). In general, histone acetylation is associated with enhanced transcriptional activity, whereas deacetylation suppresses gene expression by promoting chromatin condensation (78). HDACs are classified into four main classes: class I (HDAC1, 2, 3, and 8), class IIa (HDAC4, 5, 7, and 9), class IIb (HDAC6 and 10), class III (SIRT1-7), and class IV (HDAC11) (76, 79).

Most studies have focused on joint tissue, particularly fibroblast-like synoviocytes (FLS), where dysregulation of the HAT/HDAC balance and increased expression of proinflammatory genes have been observed (80–82). Although early studies of histone modifications in rheumatoid arthritis focused predominantly on synovial tissue and fibroblast-like synoviocytes, increasing attention has been directed toward peripheral blood-based material, particularly PBMCs, owing to their accessibility, minimal invasiveness, and suitability for biomarker development and longitudinal monitoring. At the same time, mechanistic studies in synoviocytes and macrophages have demonstrated that aberrant histone acetylation promotes excessive production of proinflammatory cytokines (TNF-α, IL-6, IL-1β) and matrix-degrading enzymes (MMPs), supporting both the pathogenic relevance of histone modifications and the therapeutic potential of HDAC inhibition in RA (83). A seminal mechanistic study by Li et al. demonstrated that PBMCs from RA patients exhibit reduced HDAC3 expression and activity, increased histone acetyltransferase activity, and significantly elevated total histone H3 acetylation compared with healthy controls, supporting the contribution of altered HDAC/HAT balance to epigenetic dysregulation in RA (84). Importantly, these epigenetic alterations were associated with enhanced expression of pro-inflammatory genes and increased activation of circulating T lymphocytes. Although this study predates the primary 2020–2025 literature window, it is repeatedly referenced by subsequent blood-based epigenetic studies and provides essential mechanistic context for interpreting later findings. For this reason, it is included as a foundational reference in the present review. Subsequent studies published within the 2020–2025 timeframe have reinforced and extended these observations. Furthermore, modulation of HAT and HDAC activity in PBMCs has been shown to downregulate chemokine receptor expression and proinflammatory cytokine production, resulting in attenuated T-cell activation and highlighting the therapeutic potential of targeting histone acetylation pathways (85).

Beyond bulk PBMC analyses, cell-type-specific epigenetic profiling has revealed additional layers of complexity in rheumatoid arthritis. Using ATAC-seq across major peripheral immune cell populations, Zong et al. demonstrated that monocytes from RA patients display substantially increased chromatin accessibility compared with lymphocyte subsets, with preferential enrichment at regulatory regions associated with antigen presentation pathways and inflammatory cytokine genes (86). This epigenetically permissive chromatin landscape likely facilitates sustained innate immune activation and enhanced cytokine output, thereby shaping an inflammatory milieu that influences downstream adaptive immune responses. Importantly, epigenetic dysregulation is not confined to innate immune compartments but also critically affects T-cell fate decisions, contributing to imbalances between regulatory T cells and Th17 effector populations that are characteristic of RA. In line with this, a prospective study of 45 RA patients at baseline, 16 RA patients post-treatment, and 17 healthy controls demonstrated using flow cytometry that dysregulated acetylation and methylation correlate with disturbed Treg/Th17 balance and increased inflammatory responses, although heterogeneous treatment regimens and limited stratification by therapy may confound these findings (87).

Finally, emerging data indicate that histone acetylation is dynamically modulated by metabolic cues. Chandrasekaran et al. (preprint) reported that insulin signaling alters histone H3 acetylation in PBMCs from RA patients, linking immune activation with metabolic regulation. Although conceptually innovative, the study remains exploratory, as detailed cohort characterization and peer-reviewed validation are not yet available, limiting its immediate clinical interpretability (88).

Collectively, studies published between 2020 and 2025, summarized in **Table 3**, demonstrate that dysregulated histone acetylation is a shared feature across multiple immune cell types in RA. These markers differ substantially in procedural feasibility, sensitivity, and translational readiness. PBMC-based H3 acetylation offers accessibility but lacks longitudinal validation; monocyte ATAC-seq provides mechanistic depth but limited practicality; Treg/Th17-associated epigenetic markers capture treatment responsiveness but are confounded by therapeutic effects; and metabolic modulation of histone acetylation remains exploratory. A critical limitation across many studies is insufficient control for treatment status. In several cohorts, patients were sampled while receiving disease-modifying antirheumatic drugs, raising the possibility that observed epigenetic signatures partially reflect pharmacodynamic effects rather than primary disease pathology. This potential bias should be explicitly acknowledged as a limitation of the field and underscores the need for studies in treatment-naïve patients and standardized longitudinal designs.

In summary, histone modifications represent both mechanistic drivers of immune dysregulation and promising biomarker candidates in rheumatoid arthritis. Future translational progress will depend on harmonized analytical methodologies, rigorous control of clinical confounders including treatment exposure and integration of epigenetic profiling with longitudinal clinical outcomes and therapeutic response metrics. Such approaches will be essential to enable reliable disease monitoring and the development of personalized therapeutic strategies.

## 7 Epigenetic regulation by non-coding RNAs in rheumatoid arthritis

### 7.1 Micro RNAs

miRNAs are small, single-stranded non-coding RNA molecules, approximately 18–25 nucleotides in length, that regulate gene expression primarily by inhibiting translation or inducing degradation of target mRNAs (89). miRNAs can simultaneously regulate multiple genes, playing a critical role in fine-tuning biological processes such as cell proliferation, differentiation, apoptosis, and immune responses (90, 91). Their regulatory action allows for dynamic and context-dependent adjustment of gene expression to meet cellular demands (89). In the immune system, miRNAs are fundamental for maintaining immune homeostasis by regulating both activation and resolution of inflammatory responses (92, 93). Dysregulation of miRNA expression plays a crucial role in the pathogenesis of RA, contributing to aberrant activation of key signaling pathways, chronic inflammation, and joint tissue destruction (94). Collectively, these features position miRNAs as integrators of genetic, epigenetic, and environmental signals, linking immune activation with chronic inflammatory persistence in RA.

Growing interest in miRNAs in RA reflects their dual relevance as mechanistically informative regulators and candidate biomarkers and therapeutic targets (95–98). In principle, disease-associated miRNA profiles could support earlier diagnosis, risk stratification, and treatment monitoring, and might inform therapeutic strategies based on miRNA modulation (99). At the same time, robust translation remains challenged by validation and standardization gaps, as well as analytical issues (e.g., batch effects, platform differences, and off-target considerations for therapeutic applications). Although bioinformatics and machine learning (ML)/artificial intelligence (AI) approaches can integrate miRNA profiles with clinical variables, their clinical utility depends on rigorous external validation and transparent evaluation of incremental value over established diagnostic and monitoring frameworks (100). Importantly, miRNA-based biomarkers are unlikely to replace established clinical indices but may provide complementary, biology-informed layers that refine diagnosis and disease monitoring.

Reported miRNA alterations span blood fractions and tissue compartments; for example, miR-155 and miR-146a are frequently described as overexpressed in PBMCs and synovial compartments, consistent with pro-inflammatory activity (92, 101). From a feasibility perspective, serum single-miRNA assays are attractive for qRT-PCR workflows, whereas plasma single markers (e.g., miR-17, miR-106b) often show only moderate discrimination, limiting standalone utility (102). Among single-marker candidates, miR-155 has been reported as overexpressed in PBMCs/serum and correlated with TNF-α and TGF-β1, with strong case–control performance (AUC 0.972; sensitivity 94.4%; specificity 88.9%) (103). Among single-marker candidates, serum miR-223 has emerged as one of the most robust diagnostic miRNAs, demonstrating high discriminatory performance between RA patients and healthy controls AUC = 0.85 (94, 104). Whole-blood miR-186-5p is decreased and associated with activity/exacerbation, although without formal diagnostic accuracy metrics (105). Additional whole-blood candidates (miR-99b-5p, miR-101-3p, miR-431-5p; **Table 5**) show promising performance in individual studies (106), and miR-103a-3p has been reported as increased in asymptomatic first-degree relatives (107). Findings for miR-21 are heterogeneous across settings (108, 109), underscoring fraction- and cohort-dependence. To capture disease heterogeneity, multi-miRNA serum panels (e.g., miR-126-3p, let-7d-5p, miR-431-3p, miR-221-3p, miR-24-3p, miR-130a-3p, miR-339-5p, let-7i-5p) can improve discrimination, with selected components achieving AUC > 0.80 (110).

For monitoring and treatment response, serum miR-21-5p and miR-142-3p correlate with inflammatory markers (TNF-α, CRP, ACPA) (109). and an MTX-response–associated panel (miR-212-3p, miR-338-5p, miR-410-3p, miR-537) has been reported in early RA (111). PBMC miR-7 (ciRS-7/miR-7 axis) remains preliminary and requires broader validation (112). Sequencing-to-qRT-PCR workflows identified plasma signatures (miR-22-3p, miR-24-3p, miR-96-5p, miR-134-5p, miR-140-3p, miR-627-5p), but limited specificity *vs* other autoimmune diseases suggests that part of the signal reflects shared inflammatory programs rather than RA-specific pathology (113). Esponse-linked markers include exosomal miR-27a-5p (AUC 0.92; sensitivity 90.6%; specificity 84.4%) and PBMC miRNAs in tofacitinib-treated, MTX-resistant RA (114), serum miR-19b-3p predicting JAKi response (AUC = 0.85) (114), and miR-210-3p with only moderate diagnostic accuracy (AUC ~0.75), supporting use mainly within composite panels (115). EV-derived miRNAs such as miR-204-5p have been validated across discovery/replication cohorts but remain technically more demanding than serum assays (116).

Overall, miRNA dysregulation is a key epigenetic layer in RA and a plausible biomarker source, yet clinical deployment will depend on standardized workflows, external validation, and clearly defined context-of-use with demonstrated incremental value (117). Detailed characteristics of individual miRNAs are summarized in **Table 4**. Thus, miRNAs should be viewed not as isolated markers but as components of integrative biomarker frameworks that reflect dynamic immune regulation across disease stages.

### 7.2 Long non-coding RNAs

lncRNAs represent a heterogeneous class of RNA molecules longer than 200 nucleotides that lack protein-coding potential yet exert broad regulatory functions at multiple levels of genomic and cellular organization (118, 119). In contrast to miRNAs, which primarily regulate mRNA stability and translation, lncRNAs operate through a wider spectrum of mechanisms, including transcriptional, epigenetic, and post-transcriptional regulation (120, 121). LncRNAs can function as molecular “scaffolds,” “decoys,” or “guides,” capable of interacting with regulatory proteins, other RNA molecules-including microRNAs-as well as specific DNA sequences (119). Through the formation of such multimolecular complexes, lncRNAs can modulate transcription factor activity, mRNA stability, and chromatin architecture, for example by recruiting histone-modifying complexes such as Polycomb Repressive Complex 2 (PRC2), thereby influencing promoter accessibility and transcriptional output (121, 122). In addition, lncRNAs contribute to higher-order genome organization by participating in chromatin domain structuring and long-range enhancer-promoter interactions. Their expression is often tissue-specific and dynamically regulated over time, supporting roles in development as well as in cellular responses to environmental and inflammatory stimuli (119).

In RA, blood-based profiling has identified numerous dysregulated lncRNAs across PBMCs, serum, whole blood, and extracellular vesicles. Mechanistically informative examples include HOTAIR, which has been linked to NF-κB signalling in a cell type– and context-dependent manner, supporting its relevance as a biomarker/target candidate rather than a unidirectional inflammation regulator (51, 123). Among more consistently reported candidates, MEG3 is described as downregulated in immune/synovial compartments and associated with disease activity, aligning with putative anti-inflammatory functions linked to NF-κB–dependent pathways (124, 125). Conversely, NEAT1 is upregulated in PBMCs and serum and linked to macrophage activation and cytokine production; its detection in circulating exosomes suggests an active role in intercellular communication (126). IFNG-AS1 is increased in PBMCs and correlates with activity through regulation of IFN-γ expression, consistent with a dual biomarker/effector role (127).

From a translational standpoint, feasibility and readiness differ by specimen and model complexity. Serum-based lncRNAs are compatible with standardized qRT-PCR workflows, whereas PBMC-derived panels and exosome-derived assays may better capture biology but require more complex pipelines and face scalability/cost constraints. Reported diagnostic candidates include a PBMC-detectable panel incorporating Linc00152/lnc-ADM-1 (AUC 0.886) (128). Several serum-derived lncRNAs demonstrate notable diagnostic performance. THRIL and HOTAIR have been reported to be differentially expressed in RA serum and to discriminate patients from healthy controls in ROC-based analyses (129, 130). These mechanistic insights are largely based on synovial tissue-derived studies and are discussed here to provide biological context for blood-based lncRNA biomarkers rather than as direct diagnostic evidence. In addition, serum ITGB2-AS1 has emerged as a promising diagnostic candidate, with combined panels incorporating ITGB2-AS1 and inflammatory markers achieving high diagnostic accuracy (131). NORAD has been proposed as a serum biomarker with AUC values >0.90 in independent cohorts (132). Combined multi-marker approaches, such as the joint assessment of KCNQ1OT1 and miR-210, may further enhance diagnostic accuracy, underscoring the potential value of integrative lncRNA-miRNA signatures (133). Notably, for some candidates, including RNA143598, the current evidence is limited to single, cross-sectional studies without independent validation, and reported diagnostic performance should therefore be interpreted cautiously.

Multi-lncRNA PBMC panels (OIP5-AS1, LINC00494, TSPOAP1-AS1, MCM3AP-AS1, LINC01588) have been reported to achieve combined AUCs >0.90 (134). Clinically relevant “add-on” models include a whole-blood composite incorporating PTPRE expression with neutrophil count and red cell distribution width, showing promise in seronegative RA (135). Exosomal lncRNAs (TCONS_I2_00013502, ENST00000363624) may add diagnostic value when combined with ACPA, although technical complexity remains a barier (136). Beyond these, additional lncRNAs (e.g., H19, TP53COR1/lincRNA-p21, SNHG3, SNHG14, GAPLINC, RNA143598, NUTM2A-AS1) are supported mainly by exploratory or single-center studies and require independent validation before clinical translation (137–145). Key lncRNAs and their proposed functions are summarized in **Table 5**.

Overall, blood-based lncRNAs constitute a complementary epigenetic biomarker layer in RA, reflecting immune dysregulation and inflammatory signalling, with potential applications in diagnosis, stratification, and therapy monitoring. However, progress toward clinical implementation will depend on standardized workflows, adequately powered longitudinal cohorts, and multi-center validation, including exploration of utility in preclinical RA settings (146–149).

### 7.3 Circular RNAs

circRNAs represent a unique subclass of endogenous non-coding RNAs characterized by a circular, covalently closed structure formed through a process known as back-splicing, in which the 3′ end of an exon is joined to the 5′ end of an upstream exon. This unusual configuration renders circRNAs relatively resistant to exonucleases and RNA decay machinery, resulting in a significantly longer cellular half-life compared to linear transcripts (150, 151). Initially considered byproducts of aberrant splicing, circRNAs are now recognized as important regulators of gene expression. One of the best-characterized mechanisms of circRNA function involves their ability to sequester microRNAs via a “miRNA sponge” mechanism, thereby modulating the availability of miRNAs and indirectly influencing the translation of their target mRNAs (152, 153). Moreover, circRNAs can function as protein-binding platforms, have been shown to modulate protein localization or activity, and in some cases form complexes with transcription factors, thereby influencing the expression of nuclear genes (154). Increasing evidence also indicates that selected circRNAs may be translated into functional peptides, although the prevalence and physiological relevance of this mechanism remain under active investigation (155, 156).

In the context of immunology and inflammatory diseases such as RA, circRNAs have been implicated in the modulation of immune responses, cell proliferation, and cytokine regulation. Altered circRNA expression profiles have been reported in serum, whole blood, and synovial tissues of RA patients, supporting their involvement in disease-associated molecular networks (157). circRNAs may contribute to the regulation of inflammatory signaling pathways, including NF-κB, JAK/STAT, and PI3K/AKT, primarily through circRNA-miRNA-mRNA regulatory axes rather than direct pathway activation (158). These synovial findings are discussed for mechanistic context rather than diagnostic interpretation. Owing to their stability, relative tissue specificity, and detectability in body fluids such as plasma and synovial fluid, circRNAs are attracting increasing interest as potential non-invasive diagnostic and prognostic biomarkers, as well as emerging therapeutic targets in autoimmune diseases (159).

Blood-based circRNAs in RA can be broadly discussed in terms of diagnostic/prognostic detectability in accessible biospecimens and association with disease activity and inflammatory burden. Notably, circNUP214 has been shown to be upregulated in peripheral blood mononuclear cells of patients with rheumatoid arthritis and to positively correlate with IL-23R expression and Th17 cell frequency, reflecting a mechanistic link between circRNA dysregulation and pathogenic Th17-driven inflammation, with moderate diagnostic performance (AUC = 0.76) but high specificity (160) (162, 163). For example, circRNAs such as hsa_circ_0000175 and hsa_circ_0008410 have been identified in the whole blood of RA patients and have been shown to correlate with disease activity indices and inflammatory markers, including C-reactive protein levels and DAS28 scores (161, 162). For hsa_circ_0000175, a significant upregulation in the serum of RA patients compared with healthy controls has been reported, along with its involvement in pyroptosis induction via the miR-31-5p/GSDME axis, suggesting its potential as a therapeutic target; however, these findings are based on a single-center functional study with a small sample size (n = 28 RA, 28 controls) rather than a diagnostic cohort (163). Several PBMC-derived circRNAs, including hsa_circ_0001200, hsa_circ_0001566, hsa_circ_0003972, and hsa_circ_0008360, have been identified as candidate diagnostic markers in RA through RNA-seq and validated by RT-qPCR, although cohort sizes were small (164). Additionally, hsa_circ_ demonstrated sex-specific diagnostic potential in female RA patients compared with healthy controls and other arthropathies, with AUCs ranging from 0.704 to 0.818 (165). Plasma circRNAs such as hsa_circ_0005008 (AUC = 0.829) and hsa_circ_0005198 (AUC = 0.783) have been associated with disease activity in newly diagnosed RA (166) while hsa_circ_101328, detected in PBMCs, exhibited high diagnostic performance (AUC = 0.957) but requires further validation in larger cohorts (167). Other circRNAs, including hsa_circ_0003353 (PBMC/serum), hsa_circ_0005567 (plasma), and exosomal hsa_circ_0003914, highlight potential functional and therapeutic roles in RA (168–170). Finally, ciRS-7, acting via the ciRS-7/miR-7 axis in PBMCs, has been implicated as a diagnostic circRNA with an AUC of 0.766, although studies are preliminary and sample sizes are limited (112).

These findings suggest that circRNAs may reflect systemic inflammatory burden rather than acting solely as disease-specific markers. A summary of the main circRNAs investigated in RA, their proposed biological functions, and reported diagnostic relevance is provided in **Table 6**.

From a translational perspective, the clinical readiness of circRNA biomarkers remains constrained by limited external validation, cohort heterogeneity, and methodological variability (e.g., differences in RNA preparation, detection platforms, and normalization strategies), which currently hampers cross-study comparability and implementation. Integration of circRNA profiling with other epigenetic layers, including microRNAs, long non-coding RNAs, and DNA methylation patterns, may offer a more comprehensive view of regulatory networks underlying RA pathogenesis. Such multi-layered approaches hold promise for the development of personalized diagnostic strategies and the identification of novel molecular targets for targeted therapeutic intervention.

### 7.4 Other classes of small non-coding RNAs

While miRNAs, lncRNAs, and circRNAs dominate the current RA literature, emerging evidence indicates that additional small non-coding RNA classes-piRNAs, siRNAs, Y-RNAs (and Y-RNA-derived fragments), snRNAs, snoRNAs, and tRNA-derived fragments (tRFs/tiRNAs)-may contribute to inflammatory regulation, immunogenicity, and stress-adaptive tissue responses in RA. At present, these RNA classes collectively extend the conceptual framework of RA pathogenesis to include RNA-protein complex-dependent immunity (Y-RNA/Ro60), splicing regulation (snRNAs), ribosome/translation-linked regulation (snoRNAs; tRFs/tiRNAs), and PIWI-associated epigenetic control (piRNAs), although the evidence base remains less mature than for miRNAs/lncRNAs/circRNAs (184). For consistency with prior sections, the subsections below distinguish RNA classes with emerging blood-based biomarker evidence (piRNAs, Y-RNA fragments, tRFs/tiRNAs) from those whose current relevance in RA is predominantly mechanistic or therapeutic (notably siRNAs as exogenous gene-silencing tools).

#### 7.4.1 PIWI-interacting RNA

piRNAs constitute a relatively recently discovered class of small ncRNAs, characterized by a length of approximately 24–31 nucleotides and the ability to specifically associate with PIWI family proteins, which belong to the larger Argonaute protein family (171, 172). The primary well-characterized function of piRNAs involves safeguarding the genome in germ cells against transposon mobilization, a process mediated through epigenetic mechanisms such as DNA methylation and the degradation of transposon transcripts (173). Beyond this pivotal role, growing evidence indicates that piRNAs are also active in somatic tissues, where they participate in the regulation of gene expression, modulation of autophagic processes, and immune responses (172).

In the context of autoimmune and inflammatory diseases such as RA, increasing attention has been directed toward the PIWI/piRNA pathway as a potential epigenetic regulator of immune responses. One of the earliest studies implicating this pathway in RA was reported by Pleštilová et al., who demonstrated altered expression of PIWI family proteins (PIWIL2 and PIWIL4) in peripheral leukocytes and synovial tissue of RA patients. Notably, PIWIL4 expression was significantly reduced in active RA and inversely correlated with inflammatory markers such as C-reactive protein (CRP) and interleukin-6 (IL-6), suggesting that PIWI-associated epigenetic mechanisms may contribute to immune dysregulation in RA (174).

Direct blood-based piRNA evidence remains exploratory. PBMC profiling identified multiple dysregulated piRNAs, with subsequent validation highlighting piR-hsa-27620 and piR-hsa-27124 as upregulated in RA PBMCs versus controls (175).

Complementary data reported increased piR-hsa-27124 in PBMCs but decreased piR-hsa-35982 in plasma, emphasizing compartment-specific regulation. The suggestion that plasma piR-hsa-35982 may be informative in seronegative RA is clinically attractive but currently preliminary (176).

From a translational perspective, current evidence suggests that piRNAs represent an early-stage and highly exploratory class of epigenetic biomarkers in RA. Compared with miRNAs, lncRNAs, and circRNAs, the available studies are limited in number, cohort size, and external validation, and robust diagnostic metrics such as ROC AUC values are largely unavailable. Moreover, methodological heterogeneity, including differences in detection platforms (RT-qPCR versus sequencing), biological material (PBMCs versus plasma), and normalization strategies, complicates cross-study comparisons (175). Importantly, most piRNA studies to date have been cross-sectional and do not consistently control for confounding factors such as treatment status, age, sex, or comorbidities. As a result, observed piRNA expression changes may partially reflect pharmacological modulation of RNA expression rather than intrinsic disease-associated regulatory changes. Consequently, while piRNAs show promise as non-invasive blood-based biomarkers, their current clinical applicability remains limited.

In summary, piRNAs constitute a novel and emerging layer of epigenetic regulation in rheumatoid arthritis. Preliminary evidence suggests a possible involvement in immune modulation and indicates a potential but still exploratory diagnostic relevance (**Table 7**).

#### 7.4.2 siRNA

siRNAs are double-stranded RNA molecules approx. 21–23 nucleotides in length that participate in the RNA interference (RNAi) mechanism, leading to the degradation of target mRNA and subsequent silencing of its expression (177, 178). In the context of RA, there is a growing body of research focused on siRNA-based therapies: a systematic review encompassing over 140 studies indicates that siRNAs have been shown, primarily in preclinical models, to precisely inhibit key inflammatory mediators (e.g., TNF-α, IL-1, IL-6) as well as critical signaling pathways (NF-κB, JAK/STAT) involved in RA (179). Although siRNAs are typically employed as experimental tools, in recent years they have become the focus of intensive translational research for RA therapy. Innovative strategies include the design of siRNAs targeting key inflammatory mediators such as TNF-α, IL-1β, or MMP-9, as well as the use of nanocarriers (e.g., liposomes, hyaluronic acid nanoparticles) enabling selective delivery to the synovial membrane and macrophages (180). Preclinical studies have demonstrated that local administration of siRNAs effectively reduces inflammatory cell infiltration, joint swelling, and levels of proinflammatory cytokines (181). The application of siRNAs in RA represents an innovative dimension- not only as an experimental research tool but also as a potential platform for targeted therapy. Incorporating this class of RNA into therapeutic development expands the scope of molecular intervention and highlights the broader translational potential of small RNAs beyond biomarker profiling.

In contrast to miRNAs, lncRNAs, and circRNAs, there is currently no robust evidence for stable basal dysregulation of endogenous circulating siRNAs that could be exploited for diagnostic purposes in rheumatoid arthritis. Endogenous siRNAs are not typically detected as stable, circulating RNA species in peripheral blood, and their physiological roles in somatic tissues remain less well defined. Consequently, siRNAs have not yet been established as blood-based biomarkers for RA diagnosis or disease stratification. Accordingly, the relevance of siRNAs in RA is currently predominantly therapeutic rather than diagnostic, as they are primarily applied as exogenously designed molecules to selectively silence pathogenic genes. This distinction underscores an important conceptual difference between siRNAs and other epigenetic RNA classes and highlights why siRNA-based approaches currently complement therapeutic development rather than biomarker profiling.

#### 7.4.3 Y-RNAs and Ro60-Y RNA complexes

Y-RNAs are small (~80–110 nt) RNAs that associate with the Ro60 protein (autoantigen SSA2) in cells to form RNP complexes. Ro60 functions as an RNA quality control factor, stabilizing Y-RNAs and participating in the cellular stress response (182). In the context of autoimmune diseases, the release of Ro60-Y RNA complexes can activate TLR7/8 receptors in antigen-presenting cells, leading to the production of proinflammatory cytokines and the breakdown of immune tolerance (182). Although specific studies on Y-RNAs in RA remain limited, an analysis of small RNA profiles in the plasma of RA patients revealed a significant reduction in the levels of Y-RNA-derived RNAs (yDRs) compared to healthy controls (183). This dual biomarker-mechanistic profile makes Y-RNAs and yDRs conceptually relevant to RA pathogenesis as well as minimally invasive monitoring. Detection of Y-RNAs and related fragments typically relies on small RNA sequencing approaches, complemented by quantitative RT-qPCR or digital PCR assays; however, their generally low abundance and the lack of standardized normalization procedures remain important limitations that need to be addressed in future studies.

#### 7.4.4 snRNAs

snRNAs are essential components of the spliceosome, which participates in pre-mRNA splicing. The major snRNA types (U1, U2, U4, U5, U6) form snRNP complexes with proteins, enabling precise intron removal and the generation of alternative protein isoforms (184). Dysfunction of snRNAs leads to aberrant splicing, which can result in the overproduction of proinflammatory proteins. Recent studies have shown that synovial cells from RA patients exhibit deregulation of the splicing machinery, including alterations in snRNA and snRNP expression, which correlate with disease activity (185). These findings suggest that snRNAs may represent a key component of post-transcriptional regulation of inflammatory genes in RA, opening new therapeutic opportunities based on splicing modulation. While current evidence is largely tissue- and mechanism-focused, these findings motivate further evaluation of snRNA-related signatures in accessible biofluids.

#### 7.4.5 snoRNAs

snoRNAs are small RNA molecules (approx. 60–300 nucleotides) involved in the chemical modification of other RNAs, primarily rRNAs, tRNAs, and snRNAs, through methylation or pseudouridylation (186). In recent years, numerous snoRNAs with extranucleolar functions have been identified, including roles in regulating mRNA stability and the expression of inflammatory genes. Reviews from 2023–2024 highlight that specific snoRNAs (e.g., SNORD72, SNORA74A) are dysregulated in inflammatory and autoimmune diseases, suggesting their involvement in controlling the translation of proinflammatory proteins (187). In RA, RNA-seq studies have revealed significant alterations in snoRNA profiles in synovial fibroblasts and leukocytes, which may reflect disruptions in translational homeostasis under chronic inflammatory conditions (183) Investigating the role of snoRNAs in RA represents an innovative aspect of research, opening avenues for the analysis of translational and epigenetic regulation (e.g., rRNA modifications in synovial cells) and their impact on the aggressiveness of synovial fibroblasts and macrophages.

#### 7.4.6 tRNA and tRNA-derived fragments

tRNAs (approx. 73–95 nucleotides) can undergo regulated cleavage under oxidative/inflammatory stress, generating tRFs and tiRNAs with regulatory roles, including Argonaute interactions and translational/cytokine modulation (188). In RA, altered circulating tsRNA/tRF patterns have been reported and associated with clinical activity, though human data remain limited (183). In rheumatoid arthritis, circulating tRFs derived from tRNA-Gly-GCC/CCC, tRNA-Glu-CTC and tRNA-Val-CAC/AAC were reported to be significantly reduced (up to three-fold) compared with healthy controls, whereas selected fragments were increased in patients with psoriatic arthritis (189). Recent reviews highlight tRFs as emerging biomarkers and potential therapeutic targets in autoimmune diseases (190).

#### 7.4.7 Synthesis and translational perspective

Synthesis and translational perspective Collectively, these small RNA classes broaden RA epigenetic/post-transcriptional biology to include siRNAs, snRNAs, snoRNAs; tRFs/tiRNAs, and Y-RNAs/Ro60, alongside piRNAs. However, compared with miRNAs/lncRNAs/circRNAs, the translational bottlenecks are more pronounced: limited external validation, cohort heterogeneity, variable pre-analytics and normalization, and incomplete confounder control (notably treatment exposure). Future work should prioritize standardized small RNA-omics pipelines, replication in independent (ideally treatment-naïve) cohorts, and integrative multi-layer models to determine whether these RNA classes add clinically actionable, context-specific value beyond established RA diagnostic and monitoring frameworks.

## 8 Limitations

Synovial tissue data are included exclusively to provide mechanistic context for circulating biomarkers and are not presented as diagnostic evidence in this blood-focused review. Several limitations should be considered when interpreting this review. First, the current evidence base on epigenetic mechanisms and biomarkers in rheumatoid arthritis is still dominated by cross-sectional studies and relatively small observational cohorts, whereas clinical trials explicitly addressing epigenetic biomarkers with hard clinical endpoints (e.g., long-term structural damage, disability, or survival) remain scarce. Accordingly, conclusions regarding diagnostic performance, prognostic value, and treatment response prediction should be regarded as hypothesis-generating and require validation in large, prospective, and clinically well-phenotyped cohorts.

Second, this work represents a structured narrative review rather than a fully systematic review with meta-analysis; no formal standardized risk-of-bias assessment or quantitative synthesis was performed. As a result, the strength of evidence attributed to individual epigenetic biomarkers depends on the design, sample size, and methodological rigor of the original studies, which are heterogeneous and not directly comparable.

Third, the included studies employed a broad range of methodological approaches, including different epigenetic platforms (from targeted assays such as quantitative methylation-specific PCR to genome-wide arrays and sequencing technologies), diverse analytical pipelines, and various biological fractions (whole blood, plasma, serum, peripheral blood mononuclear cells, and specific immune cell subsets). This heterogeneity limits the feasibility of quantitative integration and may contribute to inconsistencies in reported effect sizes, cut-off values, and directions of change for some biomarkers.

Fourth, an important limitation of the current literature is the potential confounding effect of ongoing pharmacological treatment. Many primary studies included patients receiving disease-modifying therapies, and treatment status was not always consistently reported or controlled for. As a consequence, some reported epigenetic signatures may primarily reflect treatment-induced modulation rather than intrinsic disease-associated regulation, underscoring the need for treatment-naïve and longitudinal cohorts.

Finally, the literature search was restricted to articles written in English, indexed in PubMed/MEDLINE, Web of Science Core Collection, and Scopus, and published between 2020 and 2025. Relevant studies published in other languages, outside this time frame, or indexed in other databases may therefore have been missed. Moreover, most studies were not fully balanced in terms of sex, reflecting the higher prevalence of rheumatoid arthritis in women. Although patient and control groups were generally matched for key demographic variables, residual confounding related to age, sex, smoking status, therapy, and blood cell composition cannot be excluded and may limit the generalizability of the findings.

## 9 Clinical studies on epigenetic alterations in RA

To identify ongoing or completed clinical studies related to epigenetic mechanisms in RA, we searched the ClinicalTrials.gov registry using the keyword “rheumatoid arthritis” (accessed in 2025) (191). The search was limited to studies registered between 2015 and 2025 and initially retrieved 1718 records tagged with “rheumatoid arthritis”. Titles and brief study descriptions were screened to identify trials that explicitly mentioned epigenetic modifications, epigenetic regulators or epigenetic biomarkers (e.g. DNA or RNA methylation, histone modifications, miRNAs or other ncRNAs). Trials not directly related to RA, studies withdrawn before enrolment and studies without any epigenetic endpoints were excluded. This process yielded seven clinical trials that focused on epigenetic alterations in RA: most evaluated changes in miRNA expression in response to therapeutic interventions, while one investigated a selective HDAC6 inhibitor. An overview of these trials is presented in **Table 8**. The limited availability of published results underscores that epigenetics in RA remains a promising yet still emerging research area requiring further comprehensive investigation.

The clinical study conducted by Adami et al. aimed, among other objectives, to determine changes in miRNA expression in PBMCs from RA patients in response to Filgotinib (192). Filgotinib is an orally administered JAK1 inhibitor that acts by suppressing JAK phosphorylation and inhibiting STAT3 activation. It is currently approved in Europe and Japan for the treatment of RA. Filgotinib demonstrates selective inhibition of JAK1-dependent cytokine signaling *in vitro* studies. It has been evaluated in phase 2 (DARWIN) and phase 3 (FINCH) clinical trials in patients with moderate to severe RA. In September 2020, the drug received marketing authorization in Japan and Europe. However, in August 2020, the U.S. Food and Drug Administration (FDA) requested additional data regarding the potential effects of filgotinib on semen parameters (193–195). To date, no data have been reported in the scientific literature concerning Filgotinib-induced alterations in miRNA expression levels in RA.

Conversely, the clinical study by Zissman et al. examined the impact of Tocilizumab treatment on miRNA expression (196). Tocilizumab is an interleukin-6 receptor antagonist that inhibits IL-6-dependent signaling, thereby reducing the expression of proinflammatory cytokines and enhancing the expression of genes associated with tissue repair in synovial fluid (197, 198). This drug has been extensively evaluated in clinical trials as part of combination therapies, administered both subcutaneously and intravenously, in patients with moderate to severe RA who exhibited an inadequate response to csDMARDs and/or bDMARDs. The studies confirmed that Tocilizumab represents an effective therapeutic option for adult patients with severe, active, and progressive RA who have not been previously treated with methotrexate. Moreover, the drug demonstrates high efficacy as a first-line biological therapy or in subsequent treatment lines for patients with moderate to severely active RA who have had an insufficient response or poor tolerance to at least one conventional synthetic DMARD (csDMARD) or TNF inhibitor (199–201). Additionally, a reduction in EMMPRIN/CD147 levels and an upregulation of circulating miR-146a-5p and miR-150-5p in plasma have been observed, resulting in a decreased angiogenic potential (202). However, no significant differences were found in the relative expression levels of miR-16, -21, -132, -146a, -150, -155, -203, -221, and -323. Only after stratifying patients into responders and non-responders based on CDAI and DAS28 scores was a significant change in miR-203 expression observed in the group treated with Tocilizumab for four months compared with the control group (203).

The only recent clinical study addressing histone modifications was a phase 2a trial sponsored by Chong Kun Dang Pharmaceutical investigating CKD-506, a selective HDAC6 inhibitor (204). It has been demonstrated that in RA, this compound suppresses the production of TNF-α and IL-6 in PBMCs derived from RA patients, as well as inhibits the production of MMP-1, MMP-3, IL-6, and IL-8 in FLS, thereby attenuating inflammatory responses and reducing arthritis severity (205).

Notably, to date, no large-scale clinical studies have been identified that specifically investigate DNA or RNA methylation in RA. An exception is the clinical trial NCT05808309 (206), which focuses on the identification of genomic biomarkers, including non-coding RNAs and DNA methylation profiles. However, this study remains at an early stage and involves several patient groups- individuals with RA with disease onset before 60 years of age, individuals over 65 years, patients with osteoarthritis without RA, and relatives of RA patients (both affected and unaffected). Consequently, it does not yet provide clinical data on the role of methylation in disease progression or treatment response.

Overall, while the topic of methylation in RA is being actively explored at the level of experimental and publication-based research, it is not yet well represented in clinical trials. In particular, there is a lack of clinical studies evaluating the effects of pharmacological treatments on methylation changes, which could yield valuable insights into therapeutic mechanisms and potential biomarkers of treatment response. This gap highlights a critical need for further targeted investigations into the epigenetic mechanisms underlying RA.

## 10 Translational relevance of epigenetic biomarkers based on reported AUC values

All reported AUC values are discussed descriptively and should not be interpreted as directly comparable across studies due to heterogeneity in analytical platforms, normalization strategies, and cohort composition. Across the epigenetic layers reviewed, reported AUC values provide a pragmatic, though incomplete, framework for assessing the translational relevance of blood-based biomarkers in RA. While AUC alone is insufficient to establish clinical utility, consistent performance above predefined thresholds, together with validation status and feasibility of blood-based assessment, allows stratification of candidates according to translational readiness.

Only a limited subset of biomarkers consistently reaches high diagnostic performance (AUC ≥ 0.85) and is supported by independent or multi-stage validation. Within DNA methylation studies, TNF-α, HIPK3, and selected CpG-based signatures involving CXCR5 and HTR2A demonstrate the most robust performance, including clinically meaningful gains in seronegative RA. Among non-coding RNAs, serum miR-223, miR-155, composite miRNA panels, and circRNA-based combined models, particularly hsa_circ_0000175/hsa_circ_0008410, show the strongest evidence of translational potential. Within the lncRNA layer, only NORAD and multi-lncRNA PBMC panels achieve comparable performance, indicating that panel-based strategies are generally superior to single-transcript approaches. A substantially larger group of epigenetic markers exhibits moderate discriminatory power (AUC ~0.70-0.85). These candidates-including individual miRNAs, selected circRNAs, and DNA methylation markers-are unlikely to function as standalone diagnostics but may provide incremental value when integrated into multivariate molecular-clinical models. Such hybrid approaches appear particularly relevant in seronegative RA, where conventional serological markers perform suboptimally. Most remaining epigenetic candidates fall within a discovery-phase category characterized by low AUC values, absence of ROC analysis, or reliance on small, cross-sectional cohorts. This group includes the majority of individual lncRNAs, piRNAs, Y-RNA-derived fragments, snoRNAs, snRNAs, and tRNA-derived fragments. While mechanistically informative, their current clinical relevance remains limited.

Interpretation of reported AUC values across studies is further constrained by methodological heterogeneity, lack of independent validation, cross-sectional designs, and insufficient control for treatment exposure. Notably, apparently high AUC values derived from small subgroup analyses-particularly in seronegative populations-should be interpreted with caution. Overall, these findings indicate that high AUC values alone do not ensure translational readiness. Epigenetic biomarkers most likely to progress toward clinical implementation are those that combine strong and reproducible diagnostic performance, feasibility in peripheral blood, and demonstrable added value beyond established serological tests. In RA, such biomarkers are currently confined to a narrow subset and are most likely to succeed as adjunctive tools addressing unmet needs, including seronegative disease, early detection, and treatment response stratification.

## 11 Translational and implementation considerations

Recent studies confirm that the economic burden of RA remains substantial in the biologics and targeted-therapy era, with pharmacotherapy representing the dominant cost component in many health systems. US national survey data (MEPS 2018-2020) demonstrate higher annual healthcare expenditures among patients with RA, largely driven by prescription medication use (207). Consistently, a systematic review and meta-analysis estimated total direct medical costs of approximately $12,509 per year for RA overall and $36,053 per year among bDMARD users (208). Importantly, cost-of-illness estimates vary considerably across countries due to differences in pricing, reimbursement policies, wage levels, and social security systems, limiting cross-study comparability (208–212).

From a clinical perspective, standardization of diagnostic methods has direct implications for both patient management and healthcare resource utilization. Harmonized laboratory assays, imaging protocols, and uniform clinical assessment tools can reduce diagnostic variability and unnecessary repeat testing, thereby supporting earlier and more accurate treatment decisions (212). Such standardization is particularly important for reliable patient stratification prior to escalation to high-cost biologic therapies (213) and for the clinical implementation of novel biomarkers, including blood-based epigenetic markers (214).

Evidence from real-world models of early rheumatology access further demonstrates that turnaround time between symptom onset, initial assessment, and specialist consultation is a critical determinant of timely diagnosis and treatment initiation. Structured telephone triage and rapid access rheumatology clinics have been shown to shorten waiting times while maintaining diagnostic accuracy, enabling earlier identification of patients with inflammatory rheumatic disease (215, 216).

Beyond these organizational strategies, the feasibility of implementing novel diagnostic and stratification tools in routine rheumatology practice depends on system-level readiness and workflow integration. Hybrid implementation-effectiveness research highlights the importance of standardized data capture, stakeholder engagement, and alignment with existing care pathways to ensure successful translation beyond specialized centers (217).

Within this translational framework, cost-effectiveness represents a critical prerequisite for the clinical adoption of any novel biomarker. In RA, established serological tests such as RF and ACPA are inexpensive, widely available, and highly standardized, and they remain integral components of current diagnostic and prognostic algorithms. In particular, ACPA testing offers high diagnostic specificity and meaningful prognostic information at relatively low cost, setting a demanding benchmark for emerging biomarkers (7, 218, 219).

In contrast, most blood-based epigenetic biomarkers rely on complex laboratory workflows, including nucleic acid extraction, specialized molecular platforms, and often computationally intensive bioinformatic analyses. These requirements substantially increase per-sample costs and limit scalability compared with routine immunoassays such as RF or ACPA (220, 221). At present, there is no evidence demonstrating that epigenetic assays provide a cost-effective alternative to established serological tests in routine diagnostic pathways. Systematic reviews of health economic evaluations of diagnostic biomarkers highlight persistent gaps in economic evidence and methodological challenges in evaluating the cost-effectiveness of biomarker tests, including limited data on long-term health outcomes and resource use (222). This review does not perform formal cost-effectiveness modelling, as such analyses would require prospective health-economic inputs and standardized clinical endpoints that are currently unavailable for most blood-based epigenetic assays in rheumatoid arthritis.

Moreover, reported diagnostic performance metrics, including AUC values, cannot be directly compared across studies or translated into a unified clinical protocol, consistent with current regulatory and biomarker qualification frameworks (223, 224). The potential value of epigenetic biomarkers may therefore lie not in replacing existing low-cost serological assays, but in addressing specific clinical niches where conventional markers perform suboptimally. Such scenarios may include seronegative RA, very early or preclinical disease stages, patient stratification prior to escalation to high-cost therapies, or prediction of treatment response. In these contexts, even relatively expensive biomarkers could become cost-effective if they lead to earlier diagnosis, improved treatment allocation, or avoidance of ineffective and costly therapies. However, formal cost-effectiveness analyses integrating analytical costs, clinical performance, and downstream healthcare utilization are currently lacking and should be incorporated into future prospective validation studies.

From a regulatory perspective, biomarkers intended for clinical use must comply with formal qualification and approval frameworks. Regulatory agencies such as the European Medicines Agency (EMA) and the U.S. Food and Drug Administration (FDA) distinguish between biomarker discovery for research purposes and biomarkers intended to support clinical or regulatory decision-making (“Qualification of novel methodologies for medicine development | European Medicines Agency (EMA),” 2020). In the European Union, the regulatory landscape has evolved substantially with the introduction of Regulation (EU) 2017/746 on *in vitro* diagnostic medical devices (IVDR), which defines requirements for analytical validity, clinical validity, and clinical utility of *in vitro* diagnostic tests, including genetic and molecular assays (Regulation (EU) 2017/746 of the European Parliament and of the Council of 5 April 2017 on *in vitro* diagnostic medical devices and repealing Directive 98/79/EC and Commission Decision 2010/227/EU (Text with EEA relevance.), 2017). Recent regulatory science literature emphasizes that epigenetic biomarkers, if intended for clinical application, must be supported by robust analytical performance data, standardized assay methodologies, and clear evidence of clinical relevance within a predefined context of use (224).

A critical limitation for blood-based epigenetic biomarkers in rheumatoid arthritis is the absence of systematic validation pipelines, Most published studies on blood-based epigenetic biomarkers in rheumatoid arthritis report statistically significant associations between specific DNA methylation patterns or non-coding RNA signatures and disease presence or activity; however, they rarely include independent validation cohorts, inter-laboratory reproducibility testing, or prospective study designs (183, 225). Regulatory frameworks clearly distinguish between biomarker discovery and biomarker qualification, the latter requiring a structured validation process linking the biomarker assay to clinically meaningful endpoints. Only a small fraction of candidate biomarkers across medical disciplines successfully complete this transition, largely due to insufficient analytical standardization, limited clinical validation, and lack of prospective evidence demonstrating clinical utility in real-world settings (223). Furthermore, the complexity of epigenetic assays poses additional challenges for regulatory acceptance. Differences in biological material (whole blood, plasma, serum, or isolated immune cell populations), pre-analytical handling, and analytical technologies (e.g., qMSP, sequencing-based approaches, or array-based platforms) significantly affect comparability between studies. These factors complicate the definition of standardized cutoff values and performance characteristics required for regulatory approval. As highlighted by regulatory guidance documents, early interaction with regulatory authorities and clearly defined validation strategies are essential to bridge the gap between exploratory epigenetic research and clinically implementable diagnostic assays (223, 224, 226).

At present, no published formal cost-effectiveness studies exist for blood-based epigenetic biomarkers in RA, although several authors note the substantial resource requirements for nucleic acid extraction, specialized molecular platforms, and computational bioinformatic analyses (220, 221). These requirements increase per-sample costs and limit scalability compared with standard serological assays such as RF or ACPA. Therefore, the potential value of epigenetic biomarkers may lie not in replacing existing low-cost serological tests, but in addressing specific clinical niches where conventional markers perform suboptimally-such as seronegative RA, very early or preclinical disease stages, patient stratification prior to escalation to high-cost therapies, or prediction of treatment response. In these contexts, even relatively expensive biomarkers could become cost-effective if they enable earlier diagnosis, improved treatment allocation, or avoidance of ineffective therapies.

Therefore, despite encouraging discovery-phase data, the translation of blood-based epigenetic biomarkers into routine clinical practice in rheumatoid arthritis remains substantially limited. This limitation reflects the absence of clearly defined regulatory and validation pathways, pronounced methodological heterogeneity, lack of standardized analytical and bioinformatic pipelines, insufficient prospective clinical studies, and unresolved regulatory and health-economic challenges. The current evidence base is dominated by observational, retrospective, or exploratory studies, frequently conducted in single centers with relatively small and heterogeneous cohorts, and rarely incorporating independent validation cohorts or prospective clinical endpoints. As a result, even epigenetic biomarkers demonstrating promising diagnostic performance metrics, such as high AUC values, sensitivity, or specificity in cross-sectional analyses, should at present be regarded as hypothesis-generating discovery-phase tools rather than clinically actionable diagnostic or prognostic assays. Bridging the substantial gap between biomarker discovery and routine clinical implementation will require coordinated, multidisciplinary efforts that integrate rigorous analytical and clinical validation, early regulatory alignment, implementation science frameworks, and formal health-economic evaluation to ensure feasibility, scalability, and clinical utility in real-world rheumatology practice.

## 12 Conclusions

Blood-based epigenetic biomarkers in rheumatoid arthritis represent a promising adjunct to current serological and inflammatory markers, as they are minimally invasive, repeatable, and mechanistically linked to immune dysregulation. In this structured narrative synthesis (63 publications, **Tables 2**-**8**) and registry review (7 clinical trials, **Table 1**), the most translationally plausible signals arise from a limited subset of candidates, notably selected DNA methylation markers (including TNF-α, CXCR5, and HIPK3) and circulating non-coding RNAs such as miR-223, miR-155, miR-146a, and the lncRNA MEG3, whereas the majority of reported biomarkers remain at the discovery stage. The principal barrier to clinical implementation is substantial heterogeneity across studies including biospecimen type, analytical platforms, normalization strategies, cohort design and frequent incomplete control for treatment exposure, which can confound epigenetic readouts. Future progress will require standardized pre-analytical procedures and reporting, multicenter prospective validation (particularly in treatment-naïve, early, or at-risk cohorts), and a clear definition of context of use (diagnosis, disease monitoring, or therapy response) to demonstrate incremental value and real-world feasibility in rheumatology care pathways.
